# Large-Scale Reassessment of In-Vineyard Smoke-Taint Grapevine Protection Strategies and the Development of Predictive Off-Vine Models

**DOI:** 10.3390/molecules26144311

**Published:** 2021-07-16

**Authors:** James W. Favell, Osei B. Fordwour, Sydney C. Morgan, Ieva Zigg, Wesley F. Zandberg

**Affiliations:** 1 Department of Chemistry, The University of British Columbia, 3247 University Way, Kelowna, BC V1V 1V7, Canada; jfavell@ualberta.ca (J.W.F.); osford2@gmail.com (O.B.F.); ieva.zigg@ubc.ca (I.Z.); 2 Department of Biology, The University of British Columbia, 1177 Research Road, Kelowna, BC V1V 1V7, Canada; scmorgan@health.ucsd.edu

**Keywords:** grapes, smoke taint, volatile phenols, glycosides, crop protection, fermentation, glycosidase, gas chromatography–mass spectrometry, yeast, wine

## Abstract

Smoke taint in wine is thought to be caused by smoke-derived volatile phenols (VPs) that are absorbed into grape tissues, trapped as conjugates that are imperceptible by smell, and subsequently released into wines as their free odor-active forms via metabolism by yeasts during fermentation. Blocking VP uptake into grapes would, therefore, be an effective way for vineyards to protect ripening grape crops exposed to smoke. Here, we re-evaluated a biofilm that had previously shown promise in pilot studies in reducing levels of smoke-derived VPs. A suite of nine free and acid-labile VPs were quantitated in Pinot Noir grapes that had been exposed to smoke after being coated with the biofilm one, seven or 14 days earlier. In contrast with earlier studies, our results demonstrated that in all cases, the biofilm treatments led to increased concentrations of both free and total VPs in smoke-exposed grapes, with earlier applications elevating concentrations of some VPs more than the later time points. Tracking VP concentrations through the grape ripening process demonstrated that some (phenol, *p*/*m*-cresol, and guaiacol) were not entirely sequestered in grapes as acid-labile conjugates, suggesting the presence of VP storage forms beyond simple glycosides. Free VPs in grapes, though a minor portion of the total, most clearly correlated with concentrations present in the resulting wines. Finally, red table grapes, available year round, were observed to replicate the effects of the biofilm treatments and were capable of transforming most VPs into acid-labile conjugates in under 24 h, indicating that they might be an effective model for rapidly assessing smoke-taint prophylactic products in the laboratory.

## 1. Introduction

Wildfires can cause losses and damages to any industry, including winemaking. To winemakers, particularly complex issues are generated by wildfire smoke carrying volatile organic compounds that are absorbed by grapes and taint the wine. The taint is perceived as unpleasant “ashy”, “burnt”, “medicinal” or “phenolic” character, and has been associated with volatile phenol (VP) derivatives [1] including guaiacol, 4-methylguaiacol, 4-ethylguaiacol, 4-ethylphenol, cresols, eugenol, and syringol. While these VPs may be detectable in grapes in their free forms (either by smell or analytical methods such as gas chromatography–mass spectrometry (GC–MS)), most are metabolized into conjugates that are released during the fermentation [2] and/or aging [3] of wine, causing the levels of free VPs to progressively increase. Although it is accepted that VPs are stored conjugated to various acid-labile mono- di- or trisaccharides [4,5,6,7]—that is, they are enzymatically glycosylated inside grape tissues [8]—this conversion is not well understood. Note that VP conjugates are non-volatile and do not possess the aroma of their free analogues. Recently, Szeto and colleagues reported on a significant lag in VP glycosylation in smoke-exposed grapes, suggestive of the presence of intermediates or alternative VP storage forms [6]. These observations are in agreement with our previous research that indicated that although VP metabolism in grape was rapid (within 1 h post-smoke exposure [7]), there was a significant discrepancy between the concentrations of acid-labile VP conjugates quantitated by GC–MS and identifiable GP glycosides as quantitated by high performance liquid chromatography–mass spectrometry (HPLC–MS) [9]. In addition, we have previously demonstrated that at least some smoke-derived VPs are stored within grapes as conjugates that are base-labile under conditions where VP glycosides were found resistant to hydrolysis [7]. Thus, ambiguity still exists concerning how VPs are stored within grapes, the extent to which VP glycosides (or other conjugates) are metabolized by yeast during the fermentation process, and the degree to which in-grape VP concentrations correlate with those detectible in wines produced from smoke-exposed grapes.

In addition to research into improving VP detection methods to enable smoke-taint risk assessments [1,10], studies with the objective of preventing the acquisition of smoky aromas in wine and berries are of practical importance to grape growers and winemakers. While progress has been made in identifying winemaking techniques [11,12,13,14] that minimize the release of VPs into wines during fermentation or remove them post-fermentation, these taint amelioration strategies still face several issues that limit their effectiveness. First, methods that remove VPs from wines such as fining or reverse osmosis may also remove desirable aroma or color compounds along with the unwanted VPs. For example, an activated carbon treatment of Pinot Noir to reduce smoke taint also reduced the color density, titratable acidity, and total phenolics [15]. Second, removal of free VPs still leaves behind the majority of the glycoside-conjugated forms which may be released as the wine ages. Likewise, after a reverse osmosis treatment of a smoke-tainted wine, the taint was observed slowly returning, possibly due to hydrolysis of glycoconjugates left in the wine [12], although we and others have demonstrated that VP glycosides are stable at wine pH [16]. Thus, the preferred strategy for smoke-taint prevention would be to protect the grapes from smoke while still on the vine. 

A few preventative measures have been explored with the goal of reducing the uptake of volatile phenols into the berry during growing and ripening. Grape leaf removal is one of the vineyard practices for improving crop quality and altering grapevine metabolism. Leaf removal after smoke exposure was found to correlate with a decrease in “ashy” aroma in a wine sensory study; however, analytical determination did not detect any significant changes in the levels of volatile phenols, and therefore the effect may be due to masking of the negative attributes by the enhancement of positive ones [11]. In-canopy sprinklers connected to smoke sensors have been used to mist the grapes during smoky conditions in an attempt to remove the smoke-derived particles from the air and “wash” the exposed berries [6]. While the sprinklers seemed to reduce the VP concentrations in berries harvested an hour after exposure, there was no difference either in the levels of smoke compounds at maturity or in the wine; the sensory perception of taint was unchanged as well. Kaolin is an inert clay mineral that has been applied on agricultural crops for insect control and heat protection. While its use on smoke-free Merlot berries did not significantly alter the levels of any aroma compounds [17], when applied as a protective film for smoke-taint prevention, kaolin application resulted in significant decrease in VP glycoconjugates in Merlot grapes, although no protective effects were observed in Sauvignon Blanc or Chardonnay berries [18], and an earlier report suggested that kaolin actually increased guaiacol concentrations [19]. Our previous pilot study explored the protective capacity of two commercially-used agricultural oils and an artificial phospholipid cuticle spray (biofilm), also approved for agriculture use, as potential prophylactics against VP absorption in response to smoke exposure [20]. During this previous experiment, we observed that the biofilm treatment one week before smoke exposure, significantly reduced the levels of smoke-derived VPs in Pinot Noir berries. 

The current study aims to expand on this preliminary investigation [20] by providing additional fieldwork to confirm the protective effects of these agro-sprays on grapes, and to follow the fate of smoke-derived VPs and their acid-labile conjugates during both the ripening process and through primary fermentation. Two considerations provided further impetus for this study. First, in our preliminary study, we were restricted to evaluating only one prophylactic treatment per vineyard, limiting the conditions that were testable in addition to being unable to account for the inherent variability of both grape and smoke chemistry, differing viticultural practices (which might influence the protective ability of the biofilm), etc. Second, it would be of great practical importance to determine the duration over which effective crop-protection persists, as this knowledge would guide the timing of biofilm application to vines before distant forest fire smoke blows into a vineyard. Accordingly, the protective biofilm was reevaluated here at three different vineyards and applied to grapes one, seven or 14 days prior to smoke exposure in order to assess the duration of protection. In an attempt to simulate (as much as practicable) a randomized block design, four different smoking enclosures were built on each vineyard, all of which were connected to a common source of smoke; these enclosures were situated as far apart from each other as possible. Concurrently, the same treatments were applied on store-bought table grapes in order to devise an off-vine model for studying smoke taint in a laboratory setting without the restrictions of season, wildfire frequency, and vineyard access. 

## 2. Results and Discussion

### 2.1. Development of a Table Grape Model for Evaluating Crop Protection against Smoke

**Figure 1:**
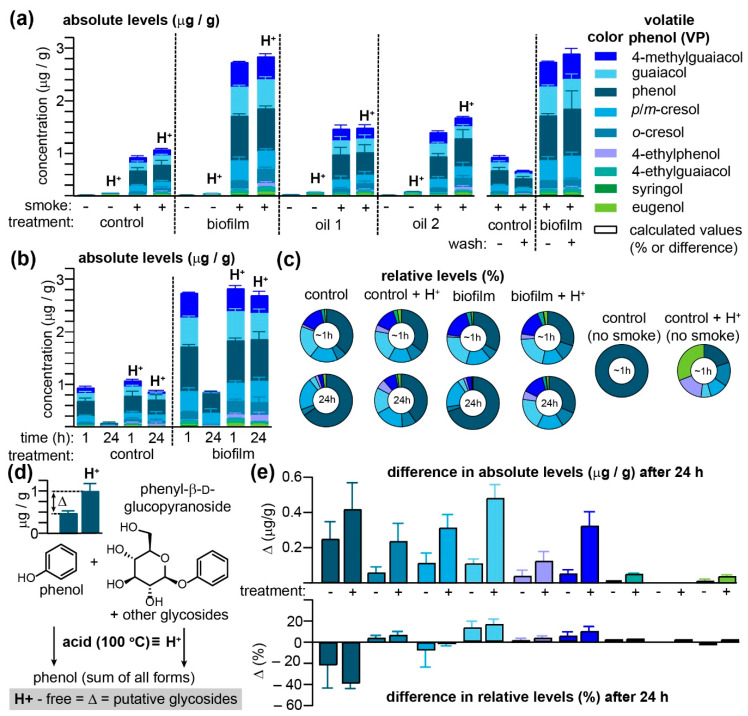
Commercial table grapes are an effective model for evaluating methods to prevent the accumulation of VPs due to smoke exposure. (**a**) Agro-spray treatments 16 h prior to smoke exposures increase the concentrations of VPs present in/on grapes processed and analyzed within one hour of smoke exposure for both untreated and biofilm-treated grapes, VPs could not be rinsed off with water. Histograms labelled with the H^+^ indicate that VPs were quantitated after acid hydrolysis. (**b**) Then 24 h after smoke exposure, both untreated and biofilm-treated table grapes had transformed significant quantities of free VPs into acid-labile conjugates, albeit (**c**) relative levels of free VPs were altered over this time period. (**d**) The difference between free and acid-labile VPs represents the fraction putatively glycosylated in-grape, illustrated here with phenol. (**e**) Differences in total (i.e., the sum of bound and free VPs) and free VPs 24 h post-smoke exposure indicate in-grape transformation; histograms depicting the difference in VP levels (Δ) are indicated with a black outline. Error bars for histograms indicate the standard error of the mean (SEM) for *n* = 3 replicate bunches of grapes except for (**e**), where they denote the quadrature-sum of the SEM.

Initial attempts by us [20] and others [16] at devising crop protection strategies to block the uptake of smoke-derived VPs into grape tissues were limited by both the number of vines that can reasonably be put into a smoke exposure apparatus (influencing the number of variables that may be tested) and the natural seasonal constraints imposed by the *Vitis vinifera* growing season (limiting the window of time available for field work). Szeto et al. [6] and Modesti et al. [13] have successfully demonstrated that wine grapes exposed to smoke immediately after harvesting replicate much of the phenotype accounting for smoke taint, i.e., VP uptake and biochemical storage as glycosides and possibly other conjugates. Accordingly, it was hypothesized that off-vine model systems would enable the rapid testing of greater numbers of protective strategies, each with more replicates, while also uncoupling these proof-of-concept tests from the need to perform experiments on active, for-profit vineyards, while simultaneously permitting year-round smoke-taint research. Commercial, imported table grapes are available in most parts of North America year round, and it is known that these varietals have the capacity to alter their biosynthesis of phenylpropanoids (including glycosylated analogues) in response to temperature changes and CO_2_ exposure [21]. While both varietal-specific and harvest-related processes undoubtedly affect the cuticle and cell wall polysaccharides of table grapes, they nevertheless afford a vineyard-like model by which the physical-chemical interactions between smoke-derived VPs, grape cuticles/cell walls, and potential prophylactic sprays may be rapidly and systematically compared. Previously, Antolini et al. [13] used table grapes to evaluate the impact of ozone treatments on smoke-taint intensity, although no attempts were made to evaluate VPs beyond guaiacol or 4-methylguaiacol, nor were VP glycosides assessed. Thus, we sought to test here whether table grapes could be used to reproduce the previously observed uptake of VPs into grapes in which they are subsequently stored as largely acid-labile conjugates, presumed to be glycosides. A parallel objective of these experiments was to reassess positive results from in-vineyard trials [20] evaluating the ability of several approved agro-sprays to block VP uptake. Accordingly, individual clusters of commercial red table grapes were treated with three previously evaluated prophylactic sprays and exposed to simulated forest fire smoke, after which nine VPs were quantitated by GC–MS/MS (Figure 1 and Supplementary Tables S1–S3), as described previously [7,10,20]. A portion of each sample was subjected to acid hydrolysis prior to VP extraction, a treatment that hydrolyzes VP glycosides, thereby permitting all VPs, whether free or bound, to be quantitated [1,6,7,10], with the difference between the two VP measurements representing the VPs transformed by grapes into glycosides and/or alternative acid-labile conjugates. 

In the absence of smoke, the free VP content in red table grapes, regardless of whether they had been treated with one of three agro-sprays, was, with a sole exception, restricted to barely detectible traces of phenol (Figure 1a and Supplementary Table S1). Subjecting samples to acid hydrolysis (H^+^), conditions which have been shown to quantitatively release simple VP glycoconjugates [10], reveals traces of *o*- and *p*- and/or m-cresol (*p*- and *m*-cresol co-eluted with the GC–MS/MS method employed), guaiacol, 4-ethylphenol, and eugenol. Interestingly, pre-treatment of grapes with oil2 (please refer to the Methods for a list of all three treatments) one day prior to smoke exposure significantly increased concentrations of these acid-labile VPs in non-smoked grapes (Supplementary Table S1). Exposure to smoke for 1 h significantly elevated VPs in all samples, with concentrations in the biofilm-treated grapes exceeding those of the control samples by over two-fold, a result that is in direct contrast with previous field studies, demonstrating that this product exhibited significant reductions in VPs after smoke exposure [20]. Oil1 and oil2 also exhibited significantly higher VP levels than control grapes, albeit not to the same level as the biofilm samples; we note that our initial evaluation of oil2 [20] also revealed that it led to increased concentrations of VPs in treated grapes. Washing the grapes prior to processing did not significantly influence the VP concentrations detectible in biofilm-treated grapes, although modest decreases were observed in untreated samples for all VPs detected (Figure 1a and Supplementary Table S2), suggesting that the biofilm in this instance was either making the grapes more porous (permitting rapid VP absorption into grape tissues and hence protection from washing) or making the grape surface more hydrophobic and hence increasing their ability to adsorb hydrophobic VPs. The inability to wash VPs off smoke-exposed grapes mimicked previous observations made by us [7] and others [6] when using on-vine wine grapes. No significant differences between free and acid-labile VPs were discernable among the four treatment groups when grapes were processed and analyzed immediately (ca. 1 h) post-smoke exposure. However, for both control and biofilm-treated grapes, significant decreases in free VPs were observed when grapes were extracted 24 h after smoke exposure (Figure 1b and Supplementary Table S3). Either slow evaporation of VP adsorbed to the grape surface or VP metabolism within grape tissues would account for these losses. The equivalence in the concentrations of acid-labile VPs in grapes processed 1 or 24 h post-smoke exposure indicates that both VP absorption into grape tissues and biotransformation into molecules with chemical properties consistent with those of VP glycosides had occurred in smoke-exposed grocery store-sourced table grapes. This transformation was somewhat slower than previously observed for on-vine berries wherein the conversion of VPs into acid-labile conjugates occurred in >1 h [7]. 

Since both control and biofilm-treated grapes were exposed to the same smoke, VP concentrations were normalized in order to deduce whether some were preferentially adsorbed to the surface of biofilm-treated grapes (Figure 1c and Supplementary Table S3). For the grapes processed and analyzed within 1 h of smoke exposure, only slight differences were observed in VP ratios between both treatment groups, independent of acid hydrolysis; this suggests that the biofilm treatment nonspecifically increased VP adsorption or absorption into table grapes. However, while VP ratios for the acid-hydrolyzed samples analyzed after 24 h closely mirrored those obtained immediately after smoke exposure, the pool of VPs remaining in their unconjugated forms after 24 h was found to be substantially enriched in phenol (rising from an average of both control and biofilm samples of 35.0% to 67.7%), at the expense of guaiacol (dropping from 20.8% to 3.8% of the total VPs over 24 h) and *o*-cresol (6.1% to 3.5%). The differences between VPs quantitated after acid hydrolysis and without hydrolysis (Δ in Figure 1d) were calculated for the control and biofilm-treated samples after 24 h (Figure 1e) as this Δ corresponds to the concentration of putative VP glycosides accrued over this period. With the sole exception of syringol, all VPs exhibited a substantial Δ at 24 h, with the biofilm-treated samples consistently exceeding untreated grapes. This transformation of free VPs into acid-labile compounds, however, appeared to be concentration independent as the Δ with respect to relative VP concentrations was essentially equivalent between both groups of grapes (Figure 1e). Note that for phenol, the large negative Δ value—which indicates that phenol was selectively enriched in the free VP pools after 24 h—was offset by relative increases in all other VPs with the exception of *p*/*m*-cresol, suggesting that some VPs (most notable for guaiacol) are selectively trapped as acid-labile conjugates within smoke-exposed table grapes. While these off-vine experiments failed to reproduce the protective effect of the biofilm with respect to VP accumulation in grapes (which might be due to numerous differences between this experiment and our prior pilot study [20]), they demonstrate that table grapes do mimic the capacity of on-vine wine grapes to sequester VPs in grape tissues primarily as acid-labile conjugates.

### 2.2. Re-Evaluation of Biofilm and Establishment of the Duration of Crop-Protection Effects

**Figure 2:**
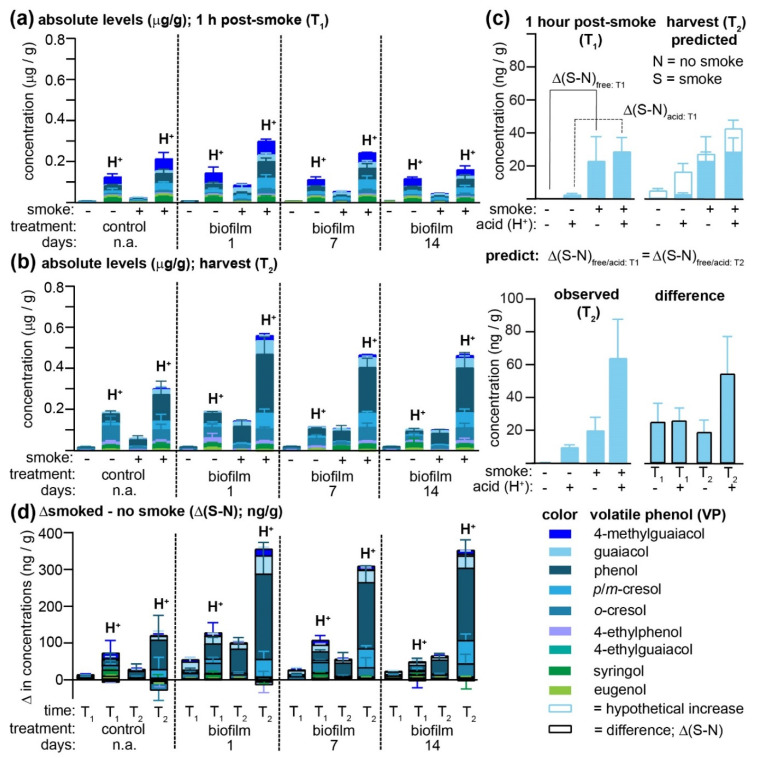
Re-evaluation of the capacity for and duration of smoke-taint protection afforded by the biofilm spray when applied one, seven or 14 days before smoke exposure at a single vineyard (vineyard 1), as compared to a spray-free control. Both free and acid-labile (H^+^) VPs were quantitated by GC–MS/MS (**a**) immediately (i.e., within 1 h; T_1_) following smoke exposure, and (**b**) at commercial maturity (T_2_). (**c**) It was hypothesized that the difference (Δ) in VP levels between grapes that had been smoke exposed (S), and non-exposed (N) controls would be equivalent at T1 and T2—as illustrated here using guaiacol concentrations in the grapes treated with biofilm 1 day prior to smoke exposure (top panel)—since all grapes were otherwise subjected to identical growing conditions until harvest. The actual Δ(S-N) for samples at harvest (black outline; lower panel), however, indicates a rise in acid-labile VP conjugates. (**d**) Acid-labile conjugates of smoke-derived phenol, *p*/*m*-cresol, guaiacol, and 4-methylguaiacol all increased as berries ripened. All error bars represent the SEM of *n* = 4 replicates except in (**d**), where they denote the quadrature-sum of the SEM.

**Figure 3:**
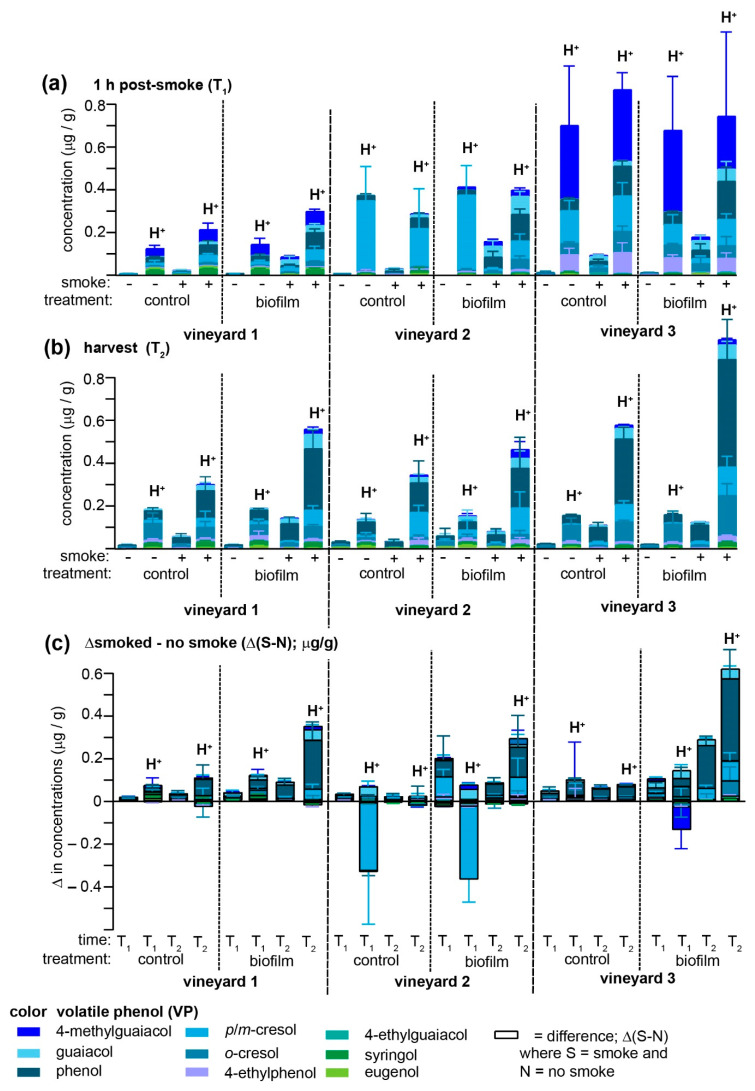
Inter-vineyard comparison of the effect of biofilm treatments on the accumulation of smoke taint-associated VPs or their conjugates in Pinot Noir grapes. Mean concentrations (*n* = 4 vines per condition) of both free and total (H^+^) VPs were determined in control or biofilm-treated Pinot Noir grapes (**a**) within 1 h of smoke exposure (T_1_) and (**b**) at harvest (T_2_); data are shown only for biofilm treatments occurring one day before smoke exposure. (**c**) Differences Δ(S-N) between the smoke-exposed (S) and non-treated (N) samples collected at each of three vineyards are depicted with histograms outlined with a black border. Error bars depict the standard error of the mean (**a**,**b**) or their sum in quadrature (**c**).

As noted, prior pilot attempts by us [20] and others [18] to evaluate smoke-taint protection strategies are limited in the number of variables that may be evaluated by the physical constraints to the number of vines that may be enclosed in a smoking apparatus in a vineyard; thus, in our prior study, the most promising treatment (biofilm) was applied at only one time point (one week) prior to smoke exposure, and to only one row of grapes in a single vineyard. To permit a more robust evaluation of this form of crop protection, and to verify the contradictory results from the off-vine study (see Section 2.1), we modified our previously described smoke-enclosure [7,20] such that four identical enclosures up to 33 m apart could be filled with smoke from a single source. Within each tent was one vine from the following four treatment groups: three biofilm spray treatments where the spray was applied either one, seven or 14 days prior to smoke exposure, and one treatment where the grapes were sprayed with water only. An equivalent set of grapevines was prepared as controls, except these were not tented nor were they exposed to the smoke. Thus, at each of three different vineyards grapes were collected from *n* = 4 replicate vines × 4 treatment groups × smoke vs. no smoke = 32 separate vines, spread over an area of ca. 33 m × 33 m. Smoke exposure for a duration of 1 h occurred at two weeks post-*veraison*, exactly as previously described, with grapes being exposed to smoke for 1.5 h. Grape samples (*n* = 4/condition/vineyard) were collected for the quantitation of both free and acid-labile VPs immediately post-smoke exposure (T_1_) and again at commercial maturity (T_2_). The time-dependent results for vineyard 1 are summarized in Figure 2a and Supplementary Tables S4 and S5; results for vineyards 2 and 3 are found in Figure 3 and Supplementary Tables S6–S9.

At vineyard 1, smoke exposure led to a significant increase in the concentrations of all free VPs (with the exception of eugenol) in non-biofilm-treated Pinot Noir grapes (Figure 2a), and concentrations of all VPs remained significantly elevated over controls at harvest (Figure 2b). In almost all cases, acid hydrolysis permitted greater concentrations of VPs to be determined for both smoke-exposed and non-exposed grapes, consistent with earlier observations that VPs are rapidly conjugated to other molecules in-grape [1,4,6,7]. An unusual exception to this trend was the widely accepted smoke-taint marker 4-methylguaiacol [22], which was found at high and essentially equivalent concentrations in all grape samples collected from vineyard 1 at T_1_ that had been subjected to acid hydrolysis (H^+^); thus, it is unlikely that this 4-methylguaiacol originated from the smoke. In agreement with the off-vine table grape experiments described above, but in contrast with the previous pilot study [20], all of the biofilm treatments led to increased concentrations of free VPs present in smoke-exposed grapes, with a significant time dependence observed for the cresols, guaiacol, and 4-ethylphenol at T_1_, and *p/m*-cresol, guaiacol, 4-methylguaiacol, 4-ethylphenol, and 4-ethylguaiacol at T_2_. More specifically, grapes to which the biofilm had been applied one day before smoke exposure had significantly higher concentrations of these VPs than those treated 14 days earlier. Likewise, the concentrations of VPs detected post-acid hydrolysis (H^+^) were higher in the biofilm-treated grapes, again exhibiting a time dependence (i.e., treatments applied earlier contained lower VPs, like the non-sprayed controls) for phenol, *p/m*-cresol, guaiacol, and 4-ethylphenol at T_1_ and phenol, guaiacol, 4-methylguaiacol, and 4-ethylguaiacol at harvest (T_2_; Supplementary Table S5). A comparison of VP concentrations (both free and total) at both time points indicates that with only a few exceptions (of low magnitude), VP levels continued to rise for both control and smoke-exposed grapes over the duration of the ripening process (compare Figure 2a,b). It was hypothesized that the difference in VP concentrations observed between smoke-exposed and control grapes (i.e., Δ(S-N), where S and N = smoked and non-smoked samples, respectively) would be equivalent in grapes analyzed immediately post-exposure (T_1_) and at harvest (T_2_; Figure 2c). This hypothesis would assume that any ripening-associated changes, hypothetically indicated for guaiacol present in one-day biofilm-treated grapes with the non-filled histograms in Figure 2c (top, right panel), would be identical for both smoke-exposed and control berries and thus that smoke-induced increases in VP concentrations would remain constant over time. While the differences in the free guaiacol concentration between smoke-exposed and control grapes did remain constant at both T_1_ and T_2_, this was not the case for the acid-labile forms (Figure 2c; bottom panel; shaded histograms with black outline), in which the concentration difference almost doubled. This increase in Δ(S-N) might indicate that smoke exposure affected in-grape biosynthetic pathways yielding acid-labile guaiacyl conjugates (which is an explanation that is inconsistent with the accepted mechanisms of feedback inhibition); alternatively, it may be due to VP sequestration in grapes as initially non-acid-labile conjugates (which are therefore unable to be detected at T_1_) which are then more slowly converted into glycoconjugates and/or other acid-labile forms over ripening (T_2_). We note that Szeto and co-workers [6] have recently provided evidence for the interconversion of in-grape VP storage forms, while Noestheden and co-workers [7] have previously documented that VPs may be trapped within Pinot Noir grapes as base-labile conjugates under conditions in which VP glycosides remain stable. These ripening-associated changes in acid-labile VPs were observed for other VPs in addition to guaiacol (Figure 2d), most notably phenol, followed by guaiacol, *m*/*p*-cresol, and 4-methylguaiacol. It is also possible that VP glycosides produced in other parts of smoke-exposed vines (e.g., leaves) may also be transported into the grapes during ripening [19,23].

Biofilm and smoke applications were evaluated at three different vineyards to provide a more robust evaluation of its potential prophylactic applications (Figure 3); for simplicity, only samples treated with biofilm one day prior to smoke exposure are depicted (but all data are summarized in Supplementary Tables S4–S9). In no case did the biofilm treatment afford protection against smoke-derived VP accumulation, and in many instances both free and acid-labile VPs in the biofilm-treated samples significantly exceeded unsprayed vines. The consistent failure to replicate crop protection with the biofilm was surprising, since one of the vineyards (vineyard 2) was the site of our initial pilot study, albeit different Pinot Noir vines were used for each experiment and obviously the fuel/smoke differed. One reason for the opposite effects of the biofilm treatment may be that the two years over which treatments were applied differed considerably with respect to total precipitation (Supplementary Table S10), with 2018 being much drier than 2019. Indeed, in all instances of biofilm treatment application in this study, rain was reported on or shortly after the date of biofilm application. Although the biofilm was initially developed to reduce instances of cuticular fractures in berries or soft fruit after rain showers [24]—which, taken in isolation might reduce uptake of VPs—the increased VP adhesiveness of biofilm-treated red table grapes (Figure 1a) and the general time-dependent decrease in this phenomenon in on-vine Pinot Noir grapes (Figure 3a,b) are consistent with the hypothesis that rain water washes away biofilm components that most strongly interact with VPs. Further experiments, including off-vine experiments, are required to reconcile these inconsistencies. 

There are several other notable observations to be made in comparing the effects of the biofilm treatments across three different vineyards. First, non-smoked grapes contained traces of free VPs but large amounts of acid-labile analogues at T_1_ (which occurred approximately two weeks post-*veraison*). However, the concentrations of the acid-labile VPs varied substantially across vineyards, with grapes at vineyard 3 containing very high amounts of 4-methylguaiacol (similar to vineyard 1) and moderate amounts of *p*/*m*-cresol and 4-ethylphenol, while non-smoked grapes at vineyard 2 possessed VP profiles dominated by *p*/*m*-cresol, exceeding 300 ng/g in all instances (Figure 3a). These high levels of (presumably) endogenous acid-labile VPs appeared to be negatively impacted by smoke exposure as a negative Δ(S-N) value was observed at T_1_ (Figure 3c) for *p*/*m*-cresol for both control and biofilm-treated vines at vineyard 2, and 4-methylguaiacol for the biofilm-treated group at vineyard 3. Interestingly, over the ripening period (T_2_), all three vineyards tended to converge toward relatively similar VP profiles (which was most noticeable among the acid-labile VPs present in smoke-exposed samples) dominated by phenol and *p*/*m*-cresol or *o*-cresol and only traces of the previously (T_1_) high 4-methylguaiacol (Figure 3b). The biofilm-treated samples from vineyards 1 and 2 closely mirrored each other in that the Δ(S-N) from both biofilm-treated and non-treated samples exhibited a clear ripening-associated increase (Figure 3c), most notable for phenol, followed by the cresols and guaiacol. 

Some practical conclusions may be drawn from this set of field studies. First, environmental conditions appear to play a significant effect on the effectiveness of potential smoke-taint prophylactics. Second, substantial amounts of endogenous VPs, and conjugates thereof, exist in *Vitis vinifera* berries, indicating that it is imperative that grape growers define a “normal” range in the absence of smoke exposure as all comparisons in the presence of smoke must be made against this significant and variable background. Third, all three vineyards exhibited substantial changes in VP concentrations over the ripening process, independent of both smoke exposure or biofilm treatment, and two vineyards clearly demonstrated ripening-associated increases in the magnitude of the difference in VP concentrations present in smoke-exposed and non-exposed grapes (most notable for the acid-labile forms). Thus, it would be most prudent for wineries to test berries for smoke taint closer to harvest rather than immediately post-smoke exposure. Finally, commercial red table grapes mimic the effects of smoke on on-vine Pinot Noir grapes, in both the adverse effects of the biofilm and in terms of their capacity to transform VPs into acid-labile conjugates, and therefore represent an adequate model to rapidly screen future prophylactic treatments. 

### 2.3. Fermentation-Associated Changes in VPs and Their Acid-Labile Conjugates

**Figure 4:**
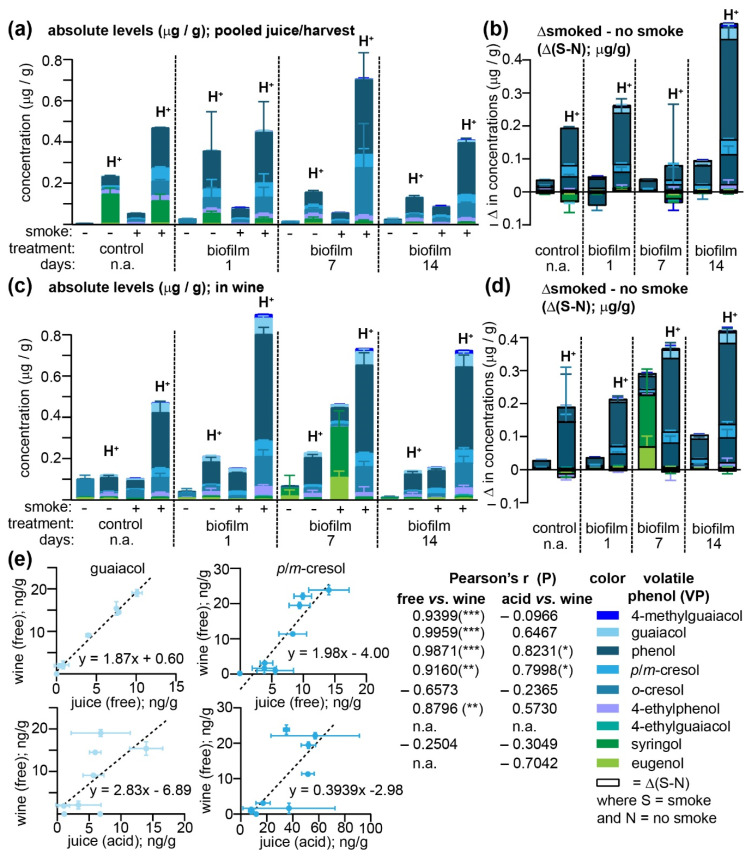
Fate of endogenous or smoke-derived, free and acid-labile VPs during the fermentation of Pinot Noir grapes subjected to differing biofilm treatments. The concentrations of both free and acid-labile (H^+^) VPs were quantitated by GC–MS in (**a**) Pinot Noir must/juice pooled from the biofilm study and (**c**) the resulting wines. Differences (Δ) in VP concentrations between the smoke-exposed (S) and non-treated (N) must (**b**,**d**) wine samples were determined and are depicted with histograms outlined with a black boarder. Error bars depict the standard error of the mean (**a**,**c**) or their sum in quadrature (**b**,**d**). (**e**) Pearson’s correlation coefficients between the concentrations of free or acid-labile VPs in the must/juice vs. the free, aroma-active forms in wines were calculated to deduce which must/juice measurement most closely correlated with the wines. Example linear regressions are only depicted for guaiacol and *p*/*m*-cresol. *, **, and *** indicate *p* < 0.05, 0.01, and 0.005, respectively.

Although the biofilm failed to protect grapes from accumulating smoke-derived VPs, at the conclusion of this field study, we were in possession of a set of samples that, due to the differing biofilm treatments, all varied with respect to the impact of their smoke exposure. When grapes from equivalent treatments across all three vineyards were pooled and fermented (representing eight pooled samples), we reasoned that this set of paired grape/must and wine samples would permit (i) an analysis of the impact of fermentation on both endogenous and smoke-derived VPs and their acid-labile conjugates and (ii) enable us to determine which measurement (free VP vs. the total) most closely predicted the actual levels of free (thus perceptible by smell) VPs in the finished wines (Figure 4 and Supplementary Tables S12 and S13). Since differing amounts of grapes remained from each treatment group and vineyard after initial analyses (Figure 2 and Figure 3), the same trends with respect to biofilm treatment increasing VP uptake more when the duration between treatment and smoking was reduced were not apparent within pooled samples (Figure 4a). Nevertheless, consistent with the VP levels detected from each vineyard at harvest (Figure 3c), phenol, followed by *p*/*m*-cresol and *o*-cresol were the most abundant of both free and acid-labile VPs detected in the eight wines; one exception to these trends was the syringol present in the control wines, which was present in high amounts in its free form. The cause of this notably apparent increase may be due to the fact that VPs from individual grape samples were quantitated in the supernatant obtained from a sample homogenate, whereas the pooled juice samples were collected immediately before inoculation and after a slow thawing process (grapes were frozen for one month prior to thawing and fermentation), followed by pressing and a 24 h cold soak to which SO_2_ had been added, i.e., the increased syringol concentration may be due to both increased duration of extraction (from grape skins) and possibly post-extraction protection from oxidation by SO_2_. The difference between smoke-exposed and non-exposed juices (Δ(S-N)) was larger for the acid-labile VPs—presumably VP glycoconjugates—than the free analogues (Figure 4b) and it was hypothesized that the magnitude of Δ(S-N) would increase for the free forms after fermentation as yeast glycosyl-hydrolases cleave these glycoconjugates [2,4]. While the concentrations of the free VPs did indeed consistently increase in all wines—in agreement with previous studies [2,6,7,20] (Figure 4c and Supplementary Tables S12 and S13)—the concentrations of the corresponding acid-labile conjugates in some instances remained essentially constant (e.g., phenol and *o*-cresol) or actually increased during fermentation; for example, total concentrations of all guaiacol conjugates in wines produced from the four smoke-exposed samples increased from a mean of 7.4 ± 1.18 ng/g in must to 52.2 ± 5.19 ng/g in the wines, while 4-methylguaiacol conjugates increased from 2.8 ± 0.70 to 14.44 ± 3.47 ng/g. Meanwhile, the Δ(S-N) for both the free and acid-labile VPs present in wines was largely unchanged with respect to the must (Figure 4d). One notable exception to this trend was the free syringol and eugenol concentrations detected in wines produced from the samples treated with the biofilm seven days prior to smoke exposure; the high concentrations of these free VPs were detected despite both the free and acid-labile quantities of these VPs in the must being below their respective method detection limits. These anomalous data for syringol and eugenol, when paired with the increase in free VPs without an accompanying decrease (and in some instances, an increase) in the total acid-labile forms suggest that, as was observed over ripening (Figure 3a,d), a fraction of VPs are sequestered as non-acid-labile conjugates and that these remaining non-acid-labile conjugates may, to some extent, be metabolized by yeast during fermentation. It is possible that these VP conjugates are present in the grape skins, which are currently not assessed by published VP quantitation methods [6,7,10,25], but are accessible to yeast during the fermentation process.

Regardless of their exact chemical form, it is clear that (i) free, odor-active VPs increased in concentration during the fermentation process and (ii) that a larger fraction of VPs found in grapes after smoke exposure were in their acid-labile rather than their free forms. Pearson’s correlation coefficients were therefore calculated between either the free or acid-labile VPs quantitated in each of the eight different must/juice samples, and the free VPs present in the resulting wines in an attempt to determine which in-must/juice measurements yielded the greatest predictive value (Figure 4e). Even though the free VPs in must/juice samples were only a minor fraction of the total, they nevertheless correlated better with the free VPs present in wines than the total acid-labile levels did; significant positive correlations were exhibited for 4-methylguaiacol, guaiacol, phenol, *p*/*m*-cresol, and 4-ethyphenol when comparing free VP levels, while only two weakly positive (but significant) correlations were observed (phenol and *p*/*m*-cresol) when total acid-labile VPs in juice were correlated against their free analogues in wines (Figure 4e). As discussed above, this lack of a clear correlation between total VPs—from which any increase in free VPs present in wine should have theoretically been attributable—would imply that either some of the free analogues in wines were originating from a conjugated VP pool that was not acid labile (and therefore unlikely to be a simple glycoside), or that the currently utilized hydrolysis methods fail to adequately detect some acid-labile VPs such as those present in grape cell walls that are omitted from the hydrolysis procedure but still included in fermentation. Further experiments are currently ongoing to investigate both of these possibilities. It is clear, however, that for the majority of VPs quantitated within this set of samples, high levels of free VPs in juice/berry samples directly correlated with high levels in wine and, based on these data, we would propose that this sole measurement of free VPs may be used by vineyards for smoke-taint risk assessment purposes, provided that the grapes are collected near commercial maturity (*vide supra*; Figure 3). Further research will be required in order to determine whether similar predictive correlations are apparent with other grape varietals, grapes grown in different regions (where fuel sources may influence VP profiles and viticulture or environmental factors may influence VP uptake or metabolism), or wines made with differing yeast strains or fermentation techniques.

In summary, our experiments to re-evaluate a biofilm that had previously exhibited the ability to prevent the in-grape accumulation of smoke-derived VPs demonstrated that this product increased VP concentrations in both red table grapes and Pinot Noir grapes grown on three different vineyards. While the source of these divergent field studies is still under investigation, with precipitation being one likely variable accounting for these discrepancies, the replication of some aspects of smoke-taint chemistry with table grapes suggests that the latter may afford a convenient way to rapidly assess how VPs interact with the grape cuticle, are biochemically transformed within grapes, and how both processes might be influenced by prophylactic treatments. Our data demonstrated that the differences in total VP levels observed between smoke-exposed and control vines on the same vineyards actually increased over the ripening process, indicating that immediately after smoke exposure, VPs might not be sequestered as acid-labile conjugates and that VP storage forms may be interconverted. We also demonstrated that endogenously occurring VPs vary considerably between vineyards (even within the same grape varietal), albeit they tend to converge at harvest, leading to the conclusion that the most accurate VP-based risk assessments (of smoke taint in wines) should utilize grape samples that are at, or close to, commercial maturity. The substantial increases in the concentrations of total (sum of both free and acid-labile) guaiacol and 4-methylguaiacol during the fermentation of smoke-exposed grapes leads us to conclude that at least some of the cryptic, non-acid-labile VPs sequestered within grapes may be metabolically accessed by yeast during the fermentation process. Nevertheless, the concentrations of free VPs in grapes, although only a minor fraction of the total, closely correlated with the final levels of the free, aroma-active VPs quantitated in the resulting wines.

## 3. Materials and Methods

### 3.1. Chemicals and General Details

Hexane, ethyl acetate, chloroform, HPLC-grade methanol (MeOH), isopropyl alcohol (IPA), hydrochloric acid (HCl), guaiacol, d_3_-guaiacol, syringol, eugenol, phenol, *o*-cresol, *p*-cresol, 4-ethylguaiacol, d_5_-4-ethylguaiacol, 4-methylguaiacol, 4-ethylphenol, and d_3_-4-ethylphenol were all purchased from Millipore Sigma (St. Louis, MO, USA) and used as received. The d_7_-*o*-cresol and d_7_-*p*-cresol internal standards (ISTDs) were purchased from Toronto Research Chemicals (Toronto, ON, Canada). All chemicals were used as received. d_3_-syringol ISTD was synthesized as reported previously [7]. Three approved agro-chemicals (two oils—oil1 and oil2—and one biofilm) known to coat berries/fruit were purchased from their respective manufactures exactly as previously described [20]. In brief, oil1 is a petroleum distillate approved for use on grapes as a fungicide, while oil2, also used in vineyards as a fungicide, consists of essential oils derived from the tea tree (*Melaleuca alternifolia*); the phospholipid-based biofilm meanwhile is approved for use to prevent the cracking of cherries or blueberries [24]. Depending on sample volume, centrifugation was performed using either an Allegra X-12R Centrifuge (Beckman-Coulter; Mississauga, ON, Canada) or a Spectrafuge 24D microcentrifuge (Mandel; Guelph, ON, Canada). An AdventurePro AV264 analytical balance (Ohaus Corporation; Pine Brook, NJ, USA) was used to prepare standards and weigh samples. A Barnstead E-Pure water purification system (Thermo Fisher Scientific; Waltham, MA, USA) was used for all water unless specifically noted. VP and ISTD stock solutions were prepared in IPA from 1 to 20 mg/L and stored at −20 ℃ for up to 12 months. Calibration standards were prepared fresh daily as per Noestheden et al. [10], with the following exceptions: the calibration range for all analytes was 1–200 ng/g; calibration samples were prepared using a 1:1 (*v/v*) hexane:ethyl acetate extract of Merlot whole-berry homogenate (prepared on a 500 mL scale) containing ISTD (50 ng/g) as the diluent. 

### 3.2. Off-Vine Study Design

To simulate the effect of smoke exposure on grape berries using an off-vine method, red Flame Seedless table grapes were purchased from a local (Kelowna, BC, Canada) grocer. One bunch of grapes was counted as a single replicate. One day prior to smoke exposure, sprays of biofilm, oil 1, and oil 2 were diluted to a concentration of 1% (*v*/*v*) and applied to the samples (*n* = 3 per sample condition) with a commercially available 1-gallon hand-sprayer (Chapin Manufacturing, Inc.; Batavia, NY, USA). Tap water was applied to the control samples in the same manner. Grapes were suspended by tying them to a metal bar with a short piece of twine so that the sprays could be evenly applied to all sides of the grapes and to ensure that no grapes were left in a puddle of the sprays. In addition to the sprayed samples and the controls, a second set of samples was sprayed with the biofilm to evaluate the effect of washing the samples with water after smoke application but prior to the sample preparation as an attempt to mimic overhead irrigation; a third set of biofilm-treated samples was prepared in identical manner to examine the effect of waiting for 24 h between smoke application and sample analysis. In all conditions, *n* = 3 replicate bunches of grapes were used. Sprays were permitted to dry for 16 h at 10 ℃ prior to smoke exposure, which is outlined in Section 3.3 below.

### 3.3. Off-Vine Artificial Smoke Application and Sample Processing

Using twine, samples were suspended from the ceiling of the modular smoke enclosure, as detailed by Noestheden et al. [7]; the grapes were approximately 1.5 m above the ground. As was performed in previous studies [7,20] and in the field studies discussed below, the fire used to produce artificial forest fire smoke was fueled by a mixture of 20% *Pinus ponderosa* needles (*w*/*w*), 30% bark (*w*/*w*; cut to 3 cm pieces), and 50% soil organic matter (*w*/*w*) collected from approximately 49.7947° N, 119.5154° W. Samples were exposed to smoke for one hour over which 1.5 kg of the fuel mixture was slowly burned with small additions of fuel at approximately 10 min intervals. Unsmoked control samples were suspended from a metal bar away from the modular smoke enclosure for the same amount of time. After smoke exposure, bunches were collected and taken for processing; there was an approximate 1 h lag between smoke exposure and the onset of sample processing. Samples were manually destemmed after collection and berries were homogenized using a cleaned Magic Bullet food processor (Homeland Housewares LLC; Los Angeles, CA, USA), centrifuged at 3000× *g* for 30 min (4 ℃) and stored at −20 ℃ before samples were analyzed with GC–MS/MS using the method developed by Noestheden et al. [10] for both free and acid-labile VPs. One set of biofilm-sprayed samples was rinsed for 30 s with cold tap water prior to processing, while a separate set of grapes (*n* = 3) was stored at room temperature for 24 h prior to processing.

### 3.4. Vineyard Studies

Pinot Noir grapevines (*Vitis vinifera*) were used for all field studies, which were conducted during the 2019 growing season at three local vineyards located near Kelowna, British Columbia, Canada: V1 (50.0588° N, 119.4335° W; elevation 467 m), V2 (49.8460° N, 119.5553° W; 420 m) and V3 (49.8110° N, 119.4911° W; 410 m). Grapevines at each vineyard were trained to vertical shoot positions with approximately 1.3 m spacing between vines. Vines were planted in north–south aligned rows at V1 and V2 while rows were west–east aligned at V3. The biofilm spray was applied to both grapes and leaves exactly as previously described [20] with the exception that the sprays were applied either one, seven or 14 days prior to smoke exposure which occurred at approximately two weeks post-*veraison* as assessed by viticulture staff at each vineyard. A fourth set of vines was sprayed with tap water only. In contrast with our previous experiments (in 2018; [20]) in which the biofilm was applied to Pinot Noir grapes during a hot growing season, 2019 was much cooler and, in several instances the biofilm was applied to grapes during or immediately preceding rain (Supplementary Table S10). At each vineyard, each of the different biofilm treatments (three time points plus the tap water-treated sample) was replicated on eight different vines that were separated from each other by at least one intervening row, such that non-adjacent samples were acquired over an area of approximately 33 m × 33 m. Four modular smoking enclosures [10,20] were constructed at each vineyard, each one housing one vine from each treatment group, i.e., one half of the all the treated samples; the remaining 16 samples (i.e., *n* = 4 for each treatment group) were not enclosed, nor exposed to smoke. Each enclosure was connected to a commercial food smoker as exactly as described above and elsewhere [7,9,20] with the exception that the smoke was diverted four ways rather than into a single smoking enclosure. Smoke was piped into each tent through 4 inch (10.16 cm) diameter flexible aluminum ducting (ProFlex; purchased at a local hardware store) typically used for venting electric clothes driers. Twenty-foot (8.8 m) sections of ducting were spliced together using rigid aluminum couplings and made gas-tight using aluminum tape, while aluminum heating duct tees were used to split the food smoker exhaust four ways. To ensure that an equal amount smoke pumped into each enclosure, the lengths of each set of tubing were kept as equivalent as possible. All grapes were exposed to smoke for 1 h and 1.5 kg of the *P. ponderosa* fuel mixture was slowly consumed over this period. Immediately after smoke exposure, several bunches of grapes (two or three depending on their size) were collected from each vine and transported to the lab, on ice, in plastic food storage bags (Ziploc^®^) for immediate processing (time point; T_1_) exactly as described above. All grapes were harvested at commercial maturity (time point 2; T_2_) as assessed by vineyard staff. Grapes were manually de-stemmed and stored in plastic food storage bags at −20 ℃ before either fermentation of GC–MS/MS analysis.

### 3.5. Fermentation

Grapes were harvested and frozen for one month until further use (as described in Section 3.4 above), at which time they were slowly thawed at room temperature over a period of 48 h. After thawing, grapes from the eight treatment groups (control vs. smoke × four biofilm applications) from each vineyard were pooled and crushed in a 20 L bladder press/hydropress (Bosagrape Winery and Brew Supplies; Burnaby, BC, Canada); all parts of the press were thoroughly rinsed with water before addition of grapes from different treatment groups. Between 20.6 and 31.7 kg of grapes were crushed and fermented for each condition. Juice plus the grape skins were collected into capped 46 L low density polyethylene primary fermentation pails (Bosagrape) that had been sanitized with a solution of 5 mg/L potassium metabisulfite (KMS) and 2.5 mg/L citric acid (both from Cellar-Tek Supplies; Kelowna, BC). SO_2_ in the form of KMS was added to all eight samples to a final concentration of 50 mg/L, after which samples were permitted to cold soak at 4 ℃ for an additional 24 h and subsequently transferred to room temperature prior to inoculation. Zymaflore RB2 (Laffort; Petaluma, CA, USA) active dry yeast and Dynastart yeast nutrient (Laffort) were both added to the must to a final concentration of 30 g/hL each following the manufacturer’s instructions. Fermentation was conducted at room temperature and the must was agitated daily; additional yeast nutrient (Laffort NutriStart) was added after nine days, and fermentation was deemed complete 13 days after inoculation. The initial and final chemical compositions of must and wine samples are recorded in Supplementary Table S11; °Brix was measured using a PAL-series pocket refractometer (Atago; Tokyo, Japan) while all other measurements were obtained using an OenoFoss (FOSS North America, Inc.; Eden Prairie, MN, USA); these metrics are recorded in Supplementary Table S12. Wines were pressed off skins using a 20 L bladder press and stored in either 23 L or 11 L glass carboys (depending on the final volume obtained) with as little headspace as possible. SO_2_ (50 mg/L) was added and the wines were stored for two months in the dark, permitting them to settle. After two months, wines were racked into 2 L glass bottles (VWR International; Radnor, PA, USA), purged with argon, and stored at 4 ℃ before further analysis.

### 3.6. Sample Analysis

VPs were quantitated exactly as previously described using GC–MS/MS [7,10,20]. In brief, a Trace 130 gas chromatograph equipped with a TSQ9000 triple quadrupole mass spectrometer (GC–MS/MS; both from Thermo Fisher Scientific; Waltham, MA, USA) was used to acquire all date in selected reaction monitoring mode. The GC column used was a Zebron ZB-5MSplus (30 m × 0.25 mm × 0.25 μm; Phenomenex; Torrence, CA, USA) and helium was used as the carrier gas (1.5 mL/min). A split ratio of 1:4 was used and 1 μL was selected as the injection volume. The GC oven and MS system parameters including all transitions were exactly as previously used [7,10,21]; see Figure S1Supplemental for representative extracted ion chromatograms for all analytes.

### 3.7. Data Analyses

Data reduction was performed using Microsoft Excel (Microsoft Corporation; Redmond, WA, USA). All statistical analyses were performed using GraphPad Prism, version 9.1.0 (GraphPad Software; San Diego, CA, USA) and R version 4.0.4 (R Core Team, R Foundation for Statistical Computing; Vienna, Austria, 2021). Details of the statistical tests performed are found in the table legends.

## Supplementary Materials

The following are available online. Table S1. Evaluation of the ability of three agro-sprays to influence the adhesiveness of VPs to table grapes; Table S2. Evaluation of the impact of washing grapes on VP levels; Table S3. Concentrations of free and total VPs in table grapes processed 1 and 24 h post-smoke; Table S4. Vineyard 1. Free VP concentrations at T_1_ and T_2_; Table S5. Vineyard 1. Total VP concentrations at T_1_ and T_2_; Table S6. Vineyard 2. Free VP concentrations at T_1_ and T_2_; Table S7. Vineyard 2. Total VP concentrations at T_1_ and T_2_; Table S8. Vineyard 3. Free VP concentrations at T_1_ and T_2_; Table S9. Vineyard 3. Total VP concentrations at T_1_ and T_2_; Table S10. Meteorological data collected for the vineyards used in biofilm field studies; Table S11. Chemical composition of must and wines; Table S12. Concentration of free VPs in must and wine samples; Table S13. Concentration of total (i.e., acid hydrolysis) VPs in must and wine samples; Figure S1. Extracted ion chromatograms for all VPs quantitated.

## Sample Availability

Samples of the compounds are not available from the authors.
