# Supplementary material for: Large-Scale Reassessment of In-Vineyard Smoke-Taint Grapevine Protection Strategies and the Development of Predictive Off-Vine Models
Source: Molecules. 2021 Jul 16;26(14):4311. doi: 10.3390/molecules26144311 (PMC8307127; doi:10.3390/molecules26144311)
Supplement: Supplementary file 1 [file molecules-26-04311-s001.zip › molecules-1291333-supplementary.pdf]

Large-scale reassessment of in-vineyard smoke-taint grapevine protection strategies and the development of predictive off-vine models

James W Favell[1,▲], Osei B Fordwour[1] , Sydney C Morgan[2,•] , Ieva Zigg[1], and Wesley F Zandberg[1,*]

[1] The University of British Columbia, Department of Chemistry, 3247 University Way, Kelowna, BC, Canada;
[2] The University of British Columbia, Department of Biology, 1177 Research Road, Kelowna, BC, Canada;
Current addresses:
▲ University of Alberta, Department of Chemistry, 11227 Saskatchewan Drive, Edmonton, AB, Canada, T6G 2G2
• Sanford Consortium for Regenerative Medicine, University of California, San Diego, 2880 Torrey Pines Scenic Drive, La Jolla, CA, USA, 92037
* Correspondence: wesley.zandberg@ubc.ca ; Tel.: +1-250-807-9821

Supplementary Materials.

Contents……………………………………………………………………………………………….....1

Table S1. Evaluation of three agro-sprays' ability to influence the adhesiveness of VPs to table grapes......2

Table S2. Evaluation of the impact of washing grapes on VP levels……………………………………..2

Table S3. Concentrations of free and total VPs in table grapes processed 1 and 24 h post-smoke……......3

Table S4. Vineyard 1: Free VP concentrations at T_1 and T_2...4

Table S5. Vineyard 1: Total (*i.e.* acid hydrolysis) VP concentrations at T_1 and T_2...5

Table S6. Vineyard 2: Free VP concentrations at T_1 and T_2...6

Table S7. Vineyard 2: Total (*i.e.* acid hydrolysis) VP concentrations at T_1 and T_2...7

Table S8. Vineyard 3: Free VP concentrations at T_1 and T_2...8

Table S9. Vineyard 4: Total (*i.e.* acid hydrolysis) VP concentrations at T_1 and T_2...9

Table S10. Meteorological data collected for the vineyards used in biofilm field studies………..……….10

Table S11. Chemical composition of must and wines……………………………………………………..11

Table S12. Concentration of free VPs in must and wine samples……………………………………………..12

Table S13. Concentration of total (*i.e.* acid hydrolysis) VPs in must and wine samples...………………….....13

Figure S1. Extracted ion chromatograms for all VPs quantitated……………………………………………14

Table S1. Evaluation of three agro-sprays' ability to influence the adhesiveness of VPs to table grapes.

smoke	treatment[1]	free	concentration (mean ± SEM) of volatile phenols (ng / g)								
			phenol[2],[3]	*o*-cresol	*p*/*m*-cresol[4]	guaiacol	4-ethylphenol	4-methylguaiacol	4-ethylguaiacol	syringol	eugenol
-	control	-	5.2 ± 0.21								
+	control	-	291.25 ± 32.34a	47.75 ± 5.95a	135.08 ± 10.31a	157.59 ± 17.92a	12.41 ± 1.29a	112.62 ± 14.92a	17.31 ± 4.14a		9.69 ± 2.36a
-	biofilm	-	5.46 ± 0.46								
+	biofilm	-	851.42 ± 22.22b	157.71 ± 4.06c	395.99 ± 37.31b	554.17 ± 18.28b	32.65 ± 6.83a	475 ± 8.25c	76.43 ± 1.79b	4.05 ± 0.46a	34.46 ± 1.83b
-	oil 1	-	5.02 ± 0.27		1.07 ± 0.04						
+	oil 1	-	479.4 ± 79.64a	90.47 ± 12.67b	188.18 ± 38.85a	274.52 ± 43.4a	15.44 ± 4.38a	228.64 ± 40.6b	34.9 ± 8.37a	2.17 ± 0.31a	17.72 ± 4.23a
-	oil 2	-	3.34 ± 0.45								
+	oil 2	-	433.83 ± 31.89a	85.53 ± 3.89b	184.28 ± 25.09a	255.43 ± 16.04a	15.61 ± 4.74a	223.01 ± 15.03ab	37.84 ± 4.4a	1.59 ± 0.58a	17.83 ± 1.94a
	S x T		****	n.a.	n.a.	n.a.	n.a.	n.a.	n.a.	n.a.	n.a.
	smoke (S)		****	n.a.	n.a.	n.a.	n.a.	n.a.	n.a.	n.a.	n.a.
	treatment (T)		****	n.a.	n.a.	n.a.	n.a.	n.a.	n.a.	n.a.	n.a.
**smoke**	**treatment**	**acid**	**phenol**	**o-cresol**	**p-m-cresol**	**guaiacol**	**4-ethylphenol**	**4-methylguaiacol**	**4-ethylguaiacol**	**syringol**	**eugenol**
-	control	+	3.63 ± 0.65	2.74 ± 0.18	2.03 ± 0.11	1.13	3.17 ± 0.58				5.69 ± 1.49
+	control	+	330.15 ± 49.87a	79.09 ± 14.6a	133.19 ± 14.68a	184.69 ± 13.75a	36.97 ± 10.17a	121.25 ± 12.59a	25 ± 4.75a		23.38 ± 4.25a
-	biofilm	+	10.78 ± 0.37		2.68 ± 0.1	1.89 ± 0.17	6.24 ± 0.7		1.44 ± 0.19		7.61 ± 0.38
+	biofilm	+	809.27 ± 27.77b	273.81 ± 19.36b	354.84 ± 20.97b	563.87 ± 27.36b	88.33 ± 15.52a	441.31 ± 30.4b	98.03 ± 1.78b	7.23 ± 0a	65.29 ± 1.42b
-	oil 1	+	15.59 ± 1.75	1.75 ± 0	3.66 ± 0.21	1.79 ± 0.13	6.96 ± 0.8		1.86 ± 0.17		7.62 ± 2.27
+	oil 1	+	436.84 ± 91.02a	134.83 ± 23.84a	172.63 ± 34.11a	270.37 ± 40.15a	40.99 ± 10.17a	212.19 ± 33.5a	43.99 ± 10.56a	3.61 ± 0.76a	36.69 ± 7.64a
-	oil 2	+	22.15 ± 3.36	3.11 ± 0	22 ± 1.76	1.66 ± 0.19	16.64 ± 5.47		3.04 ± 0.44		12.45 ± 2.02
+	oil 2	+	539.43 ± 95.65ab	179.54 ± 23.86ab	1.13 ± 0ab	234.91 ± 17.68a	79.27 ± 16.44a	172.75 ± 11.6a	53.45 ± 6.69a	3.66 ± 0.54a	42.32 ± 5.59ab
	S x T		**	-	****	****	ns	-	-	-	***
	smoke (S)		****	-	****	****	****	-	-	-	****
	treatment (T)		**	-	****	****	*	-	-	-	***

Notes: [1]All treatments are labelled identically to our previous research on the same products. Oil 1 and 2 are briefly described in the materials and methods; further details may be obtained in Favell *et al*. (2019). [2]All averages are reported ± the standard error of the mean of a maximum of three replicates; a pooled sample made from one unique cluster of grapes was considered to be a replicate. No zeros were imputed and blanks indicate that VP was below the method detection limit (MDL) for all three replicates of a particular treatment. Different letters (within columns) indicate a statistically significant difference between means of only the smoked samples for each VP as assessed using a one-way ANOVA followed by a Tukey HSD *post hoc* test ($P < 0.05$). A two-way ANOVA was used to evaluate the interaction between smoke (S) and treatment (T); *, **, *** and **** denote significant differences at $p \leq 0.05$, $p \leq 0.01$, $p \leq 0.001$ and $p \leq 0.0001$, respectively; n.s. = not significant; n.a. = not applicable (due to insufficient replicates). [4]*p*- and *m*-cresol were unresolvable by the GC-MS/MS conditions employed and the sum of these VPs is reported.

Table S2. Evaluation of the impact of washing grapes on VP concentrations[(1)].

smoke	treatment	wash[(2)]	concentration (mean ± SEM) of volatile phenols (ng / g)								
			phenol	*o*-cresol	*p*/*m*-cresol	guaiacol	4-ethylphenol	4-methylguaiacol	4-ethylguaiacol	syringol	eugenol
+	control	-	291.2 ± 32.34	47.7 ± 5.95	135.1 ± 10.31	157.6 ± 17.92	12.4 ± 1.29	112.6 ± 14.92	17.3 ± 4.14		9.7 ± 2.36
+	control	+	191.7 ± 21.87****	31.5 ± 2.81ns	91.7 ± 9.87ns	95.3 ± 8.01***	8.2 ± 1.31ns	66.9 ± 6.32ns	8.8 ± 1.32ns		4.1 ± 0.77ns
+	biofilm	-	851.4 ± 22.22	157.7 ± 4.06	396.0 ± 37.31	554.2 ± 18.28	32.6 ± 6.83	475.0 ± 8.25	76.4 ± 1.79	4.1 ± 0.46	34.5 ± 1.83
+	biofilm	+	911.8 ± 65.23ns	162.5 ± 15.57ns	458.5 ± 40.68ns	568.3 ± 53.69ns	46.8 ± 13.75ns	482.8 ± 55.68 ns	81.0 ± 12.98 ns	5.7 ± 0.72 ns	37.8 ± 7.78ns

Notes: [(1)]Only concentrations of free VPs were recorded. [(2)] + denotes samples that were washed under cold water for 30 s before processing and VP extraction. The impact of washing was evaluated using an unpaired t-test with the Holm-Sidak method to check for significance**;** *, **, *** and **** denote significant differences at $p \leq 0.05$, $p \leq 0.01$, $p \leq 0.001$ and $p \leq 0.0001$, respectively; ns = not significant.

Table S3. Concentrations of free and total (*i.e.* acid-labile plus free) VPs in grapes processed 1 and 24 h after smoke-exposure.

hour[(1)]	treatment	acid	concentration (mean ± SEM) of volatile phenols (ng / g)								
	smoke		phenol	*o*-cresol	*p*/*m*-cresol	guaiacol	4-ethylphenol	4-methylguaiacol	4-ethylguaiacol	syringol	eugenol
1	control	-	291.2 ± 32.34	47.7 ± 5.95	135.1 ± 10.31	157.6 ± 17.92	12.4 ± 1.29	112.6 ± 14.92	17.3 ± 4.14		9.7 ± 2.36
24	control	-	42.0 ± 15.20**	2.5 ± 0.28**	12.7 ± 1.61***	2.5 ± 0.71**	1.3 ns	1.8 ± 0.33**			1.3 ns
1	control	+	330.2 ± 49.87	79.1 ± 14.60	133.2 ± 14.68	184.7 ± 13.75	37.0 ± 10.17	121.3 ± 12.59	25.0 ± 4.75		23.4 ± 4.25
24	control	+	292.0 ± 53.44 ns	63.3 ± 17.94 ns	127.2 ± 32.27 ns	114.9 ± 13.60 ns	42.5 ± 18.65 ns	56.9 ± 11.65 ns	9.4 ± 1.54 ns		15.2 ± 4.91 ns
1	bioflim	-	851.4 ± 22.22	157.7 ± 4.06	396.0 ± 37.31	554.2 ± 18.28	32.6 ± 6.83	475.0 ± 8.25	76.4 ± 1.79	4.1 ± 0.46	34.5 ± 1.83
24	bioflim	-	381.2 ± 27.20***	16.1 ± 2.06****	96.3 ± 16.47**	20.8 ± 2.86****	9.4 ± 1.40*	17.4 ± 3.12****	3.1 ± 0.63****		2.2 ± 0.49****
1	bioflim	+	809.3 ± 27.77	273.8 ± 19.36	354.8 ± 20.97	563.9 ± 27.36	88.3 ± 15.52	441.3 ± 30.40	98.0 ± 1.78	7.2	65.3 ± 1.42
24	bioflim	+	801.3 ± 81.56 ns	254.7 ± 57.74 ns	411.8 ± 39.08 ns	504.2 ± 43.32 ns	136.0±30.04 ns	343.7 ± 45.15 ns	56.1 ± 1.37****	8.7 ± 0.37	41.8 ± 3.71 ns
	**smoke**		**relative concentrations (mean ± SEM) of volatile phenols (% of total)**								
1	control	-	37.2 ± 0.10	6.1 ± 0.14	17.2 ± 1.05	20.1 ± 0.23	1.6 ± 0.15	14.4 ± 0.60	2.2 ± 0.31		1.2 ± 0.18
24	control	-	65.6 ± 12.15	3.8 ± 0.75	19.8 ± 8.85	3.9 ± 0.49	2.0	2.8 ± 0.39			2.0
1	control	+	35.4 ± 0.86	8.5 ± 0.50	14.3 ± 0.39	19.8 ± 1.37	4.0 ± 0.64	13.0 ± 0.40	2.7 ± 0.17		2.5 ± 0.17
24	control	+	40.5 ± 1.46	8.8 ± 0.98	17.6 ± 1.54	15.9 ± 3.31	5.9 ± 1.50	7.9 ± 1.99	1.3 ± 0.19		2.1 ± 0.33
1	bioflim	-	33.0 ± 0.08	6.1 ± 0.88	15.3 ± 0.16	21.5 ± 0.22	1.3 ± 0.46	18.4 ± 0.07	3.0 ± 0.01	0.2 ± 0.06	1.3 ± 0.11
24	bioflim	-	69.8 ± 1.99	2.9 ± 0.11	17.6 ± 1.77	3.8 ± 0.20	1.7 ± 0.15	3.2 ± 0.30	0.6 ± 0.07		0.4 ± 0.06
1	bioflim	+	30.0 ± 0.77	10.1 ± 0.64	13.1 ± 0.58	20.9 ± 1.13	3.3 ± 0.55	16.3 ± 1.20	3.6 ± 0.07	0.3 ± 0.09	2.4 ± 0.04
24	bioflim	+	31.3 ± 1.69	10.0 ± 1.75	16.1 ± 0.87	19.7 ± 2.64	5.3 ± 0.95	13.4 ± 2.39	2.2 ± 0.08	0.3 ± 0.02	1.6 ± 0.07
	**no smoke**		**relative concentrations (mean ± SEM) of volatile phenols (% of total)**								
1	control	-	100.0 ± 0.00								
1	control	+	19.7 ± 0.65	14.9 ± 0.65	11.0 ± 0.11	6.1	17.2 ± 0.58				31.0 ± 1.42

Notes: [(1)] Hours post-smoke exposure. The difference in the VP presence over time was also evaluated by **unpaired t-test and the Holm-Sidak** method**;** *, **, *** and **** denote significant differences at $p \leq 0.05$, $p \leq 0.01$, $p \leq 0.001$ and $p \leq 0.0001$, respectively; ns = not significant.

Table S4. Vineyard 1: Free VP concentrations immediately after (T_1) smoke-exposure and at harvest (T_2).[(1)(2)(3)]

VP	smoke	**biofilm application:** days before smoke exposure				S x T	smoke (S)	treatment (T)
		**control**	**1**	**7**	**14**			
				**time:** 1 hour post-smoke exposure (T_1)				
syringol	-							
syringol	+							
phenol	-	2.1 ± 0.51	1.38 ± 0.48	1.54 ± 0.24	1.44 ± 0.16	ns	***	ns
phenol	+	4.22 ± 1.66a	10.58 ± 2.87a	7.56 ± 2.53a	7.35 ± 2.47a			
p/*m*-cresol	-	0.46 ± 0.12	0.3 ± 0.16	0.28 ± 0.12	0.25 ± 0.18	**	****	**
p/*m*-cresol	+	1.69 ± 0.51a	7.98 ± 1.38ab	5.11 ± 0.79ab	4.43 ± 0.74b			
o-cresol	-	1.31 ± 0.38	1.29 ± 0.13	1.47 ± 0.17	1.23 ± 0.32	**	****	**
o-cresol	+	4.81 ± 1.15a	18.08 ± 3ab	12.24 ± 1.75ab	10.06 ± 2.02b			
guaiacol	-							
guaiacol	+	3.91 ± 1.71a	25.15 ± 6.25ab	14.96 ± 4.1ab	10.27 ± 1.87b			
eugenol	-	0.58 ± 0.41	0.74 ± 0.16	1.23 ± 0.7	1.21 ± 0.31	ns	ns	ns
eugenol	+	1.07 ± 0.19a	1.6 ± 0.5a	0.98 ± 0.13a	1.18 ± 0.21a			
4-methylguaiacol	-	0.53 ± 0	0.1 ± 0					
4-methylguaiacol	+	1.06 ± 0.42a	12.07 ± 2.94a	5.78 ± 1.09ab	4.12 ± 0.65b			
4-ethylphenol	-	0.27 ± 0.01	0.25 ± 0.04	0.27 ± 0.03	0.24 ± 0.02	*	****	*
4-ethylphenol	+	0.44 ± 0.03a	0.83 ± 0.12ab	0.65 ± 0.05ab	0.63 ± 0.07b			
4-ethylguaiacol	-							
4-ethylguaiacol	+							
				**time:** harvest (T_2)				
syringol	-							
syringol	+	0.31 ± 0.03a	0.27 ± 0.02a	0.29 ± 0.05a	0.26 ± 0.01a			
phenol	-	11.32 ± 0.91	13.28 ± 0.65	13.14 ± 0.86	13.36 ± 0.49	ns	****	ns
phenol	+	34.42 ± 9.92a	83.91 ± 15.78a	53.73 ± 16.38a	55.96 ± 9.22a			
p/*m*-cresol	-	1.44 ± 0.11	1.85 ± 0.15	1.99 ± 0.09	1.71 ± 0.07	**	****	**
p/*m*-cresol	+	4.93 ± 1.17a	11.17 ± 1.34ab	7.55 ± 1.64b	11.05 ± 1.07b			
o-cresol	-	5.97 ± 1.09	3.57 ± 0.3	5.14 ± 1.27	4.33 ± 0.29	ns	****	ns
o-cresol	+	11.43 ± 3.28a	18.09 ± 1.98a	12.75 ± 2.6a	16.99 ± 1.86a			
guaiacol	-	1.04 ± 0.03	1.3 ± 0.08	1.31 ± 0.04	1.15 ± 0.04	*	****	*
guaiacol	+	6.92 ± 2.5a	20.41 ± 4.11ab	11.68 ± 2.17ab	13.57 ± 1.54b			
eugenol	-	0.15 ± 0	0.41 ± 0.06	0.75 ± 0.62	0.37 ± 0.11	ns	***	ns
eugenol	+	0.85 ± 0.1a	1.17 ± 0.18a	0.92 ± 0.11a	1.44 ± 0.25a			
4-methylguaiacol	-	0.4 ± 0.02	0.42 ± 0.01	0.43 ± 0.02	0.41 ± 0.01	**	****	**
4-methylguaiacol	+	1.36 ± 0.39a	5.2 ± 1.14ab	2.93 ± 0.35ab	3.4 ± 0.37b			
4-ethylphenol	-	1.39 ± 0.07	1.44 ± 0.06	1.59 ± 0.07	1.56 ± 0.04	ns	****	*
4-ethylphenol	+	1.89 ± 0.07a	2.06 ± 0.1a	2.1 ± 0.17a	2.2 ± 0.09a			
4-ethylguaiacol	-	0.19 ± 0.01	0.2 ± 0	0.19 ± 0	0.2 ± 0.02	**	****	**
4-ethylguaiacol	+	0.25 ± 0.02a	0.58 ± 0.11ab	0.36 ± 0.02ab	0.49 ± 0.05b			

Notes: [(1)] Mean VP concentrations (n = 4) are recorded in ng/g ± the standard error of the mean (SEM). [(2)] The effects of smoke (S), biofilm treatments (T) and S x T interaction were tested with a two-way ANOVA; *, **, *** and **** denote significant differences of $p \leq 0.05$, $p \leq 0.01$, $p \leq 0.001$ and $p \leq 0.0001$, respectively; ns = not significant ($p \geq 0.05$). [(3)] Different letters (within the rows) indicate the differences in effects of time of biofilm application on the smoked sample only using one-way ANOVA followed by a Tukey's HSD test. Blank cells denote concentrations below the method limit of detection.

Table S5. Vineyard 1: Total (*i.e.* quantitated after acid hydrolysis) VP concentrations immediately after (T_1) smoke-exposure and at harvest (T_2).[1][2]

VP	smoke	**biofilm application:** days before smoke exposure				S x T	smoke (S)	treatment (T)
		**control**	**1**	**7**	**14**			
**time:** 1 hour post-smoke exposure (T_1)								
syringol	-	13.17 ± 1.98	18.21 ± 3.26	11.74 ± 1.77	11.34 ± 1.27	ns	ns	ns
syringol	+	19.64 ± 1.88a	17.99 ± 3.77a	18.21 ± 1.52a	11.46 ± 2.34a			
phenol	-	23.78 ± 2.56	24.25 ± 4.54	21.73 ± 2.3	18.52 ± 1.82	ns	****	*
phenol	+	42.7 ± 8.99a	70.59 ± 7.68a	49.29 ± 10.1a	36.48 ± 9.53a			
p/*m*-cresol	-	4.96 ± 1.3	5.72 ± 0.86	5.88 ± 0.39	5.47 ± 0.45	*	****	**
p/*m*-cresol	+	10.52 ± 1.22a	20.61 ± 2.05a	17.24 ± 2.87a	10.47 ± 2.73a			
o-cresol	-	13.96 ± 5.54	15.57 ± 3.52	13.98 ± 1.14	16.2 ± 2.33	ns	****	ns
o-cresol	+	31.99 ± 3.84a	48.15 ± 7.57a	45.03 ± 8.75a	28.48 ± 5.65a			
guaiacol	-	4.19 ± 0.53	3.08 ± 0.47	2.99 ± 0.31	2.66 ± 0.14	**	****	*
guaiacol	+	12.1 ± 2.84ab	29.24 ± 4.35a	24.31 ± 4.39ab	13.97 ± 3.23b			
eugenol	-	8.16 ± 2.66	7.43 ± 1.79	7.37 ± 2.03	9.5 ± 1.71	ns	ns	ns
eugenol	+	10.52 ± 1.59a	6.28 ± 1.07b	8.03 ± 0.98ab	5.42 ± 0.69ab			
4-methylguaiacol	-	31.92 ± 7.99	44.35 ± 12.76	27.32 ± 6.45	33.87 ± 3.82	ns	ns	ns
4-methylguaiacol	+	50.28 ± 15.72a	57.8 ± 4.88a	41.63 ± 1.45a	27.75 ± 8.62a			
4-ethylphenol	-	6.6 ± 0.33	6.6 ± 1.39	4.58 ± 0.9	4.85 ± 0.29	ns	ns	*
4-ethylphenol	+	6.04 ± 0.42a	7.11 ± 0.21a	5.97 ± 0.53a	5.3 ± 0.6a			
4-ethylguaiacol	-	1.93 ± 0.07	1.46 ± 0.32	1.51 ± 0.12	1.68 ± 0.11	ns	**	ns
4-ethylguaiacol	+	1.97 ± 0.21a	2.35 ± 0.1a	2.16 ± 0.22a	1.76 ± 0.14a			
**time:** harvest (T_2)								
syringol	-	19.57 ± 2.14	22.38 ± 2.8	18.1 ± 0.75	26.81 ± 12.51	ns	ns	ns
syringol	+	24.28 ± 3.93a	21.31 ± 1.86a	24.92 ± 1.45a	20.98 ± 2.26a			
phenol	-	48.56 ± 7.65	54.58 ± 4.38	40.71 ± 3.25	21.3 ± 7.82	*	****	*
phenol	+	133.02 ± 32.78a	290.43 ± 42.95a	226.6 ± 26.15a	221.16 ± 38.2a			
p/*m*-cresol	-	16.76 ± 4.2	18.83 ± 4.5	12.04 ± 0.64	7.05 ± 2.94	ns	****	ns
p/*m*-cresol	+	44.01 ± 16.42a	74.2 ± 10.06a	68.81 ± 5.08a	74.01 ± 8.82a			
o-cresol	-	77.34 ± 20.71	51.18 ± 8.43	37.54 ± 8.9	22.92 ± 8.89	ns	ns	ns
o-cresol	+	55.51 ± 13.46a	58.7 ± 4.52a	65.76 ± 12.02a	61.2 ± 10.78a			
guaiacol	-	9.59 ± 1.19	9.6 ± 0.87	8.85 ± 0.48	9.05 ± 1.42	*	****	*
guaiacol	+	23.67 ± 7.11a	64.24 ± 11.91ab	47.27 ± 5.22ab	45.96 ± 6.81b			
eugenol	-	4.74 ± 0.97	8.22 ± 1.94	5.31 ± 1.56	6.95 ± 1.11	ns	ns	ns
eugenol	+	4.67 ± 0.66a	6.51 ± 0.89a	7.08 ± 0.35a	8.25 ± 1.18a			
4-methylguaiacol	-	1.08 ± 0.2	0.98 ± 0.23	1.06 ± 0.19	0.98 ± 0.17	**	****	**
4-methylguaiacol	+	5.35 ± 2.4a	23.91 ± 4.95ab	16.21 ± 1.56ab	15.72 ± 3.37b			
4-ethylphenol	-	14.11 ± 3.52	26.02 ± 10.54	10.62 ± 1.29	4.95 ± 2.54	ns	ns	ns
4-ethylphenol	+	11.57 ± 3.35a	15.18 ± 2.15a	13.87 ± 1.17a	15.55 ± 5.94a			
4-ethylguaiacol	-	2.59 ± 0.18	2.92 ± 0.36	2.23 ± 0.16	1.79 ± 0.8	*	****	*
4-ethylguaiacol	+	2.35 ± 0.28a	4.52 ± 0.52b	4.09 ± 0.29b	4.08 ± 0.2b			

Notes: [(1)] Mean VP concentrations (n = 4) are recorded in ng/g ± the standard error of the mean (SEM). [(2)] The effects of smoke (S), biofilm treatments (T) and S x T interaction were tested with a two-way ANOVA; *, **, *** and **** denote significant differences of $p \leq 0.05$, $p \leq 0.01$, $p \leq 0.001$ and $p \leq 0.0001$, respectively; ns = not significant ($p \geq 0.05$). [(3)] Different letters (within the rows) indicate the differences in effects of time of biofilm application on the smoked sample only using one-way ANOVA followed by a Tukey's HSD test. Blank cells denote concentrations below the method limit of detection.

Table S6. Vineyard 2: Free VP concentrations immediately after (T_1) smoke-exposure and at harvest (T_2).[1][2]

VP	smoke	**biofilm application:** days before smoke exposure				S x T	smoke (S)	treatment (T)
		**control**	**1**	**7**	**14**			
**time:** 1 hour post-smoke exposure (T_1)								
syringol	-							
syringol	+				0.09 ± 0			
phenol	-	0.15 ± 0	0.32 ± 0.18	1.29 ± 0.81	0.13 ± 0	ns	**	ns
phenol	+	11.83 ± 6.39a	59.06 ± 15a	52.29 ± 14.99a	33.14 ± 9.23a			
p/*m*-cresol	-	0.18 ± 0.15	0.33 ± 0.12	0.41 ± 0.06	0.35 ± 0.17	ns	****	ns
p/*m*-cresol	+	4.54 ± 1.49a	18.23 ± 3.34a	16.86 ± 2.43a	17.36 ± 6.7a			
o-cresol	-	1 ± 0.23	0.82 ± 0.07	0.99 ± 0.13	1.01 ± 0.28	ns	****	ns
o-cresol	+	3.45 ± 1.52a	15.85 ± 3.02a	14.69 ± 2.22a	15.17 ± 5.49a			
guaiacol	-							
guaiacol	+	13.35 ± 3.63a	55.94 ± 13.03a	45.3 ± 4.95a	48.11 ± 18.51a			
eugenol	-	0.41 ± 0.03	0.34 ± 0.27	0.63 ± 0.17	0.44 ± 0.08	ns	ns	ns
eugenol	+	0.2 ± 0.07a	0.92 ± 0.3a	1.01 ± 0.18a	0.61 ± 0.07a			
4-methylguaiacol	-	0.06 ± 0.03	0.25 ± 0.12	0.17 ± 0.08	0.11 ± 0.07	*	****	*
4-methylguaiacol	+	4.19 ± 1.12a	26.21 ± 5.75a	21.44 ± 1.57a	23.43 ± 8.56a			
4-ethylphenol	-	0.64 ± 0.04	0.66 ± 0.04	0.67 ± 0.13	0.67 ± 0.05	ns	****	ns
4-ethylphenol	+	1.34 ± 0.16a	2.71 ± 0.45a	2.55 ± 0.21a	2.72 ± 0.89a			
4-ethylguaiacol	-							
4-ethylguaiacol	+	0.5 ± 0.24a	3.67 ± 0.78a	3.02 ± 0.29a	3.74 ± 1.39a			
**time:** harvest (T_2)								
syringol	-	1.11 ± 0.18	0.94 ± 0.02	0.92 ± 0.01	0.89 ± 0.01	ns	*	ns
syringol	+	0.89 ± 0.06a	0.8 ± 0.02a	0.83 ± 0.04a	0.8 ± 0.01a			
phenol	-	14.33 ± 3.94	28.78 ± 19.34	28.16 ± 18.74	27.21 ± 10.14	ns	ns	ns
phenol	+	28.42 ± 8.54a	46.68 ± 15.95a	50.75 ± 16.29a	46.43 ± 21.06a			
p/*m*-cresol	-	2.94 ± 0.82	2.11 ± 0.17	7.01 ± 4.79	6.8 ± 2.54	ns	*	ns
p/*m*-cresol	+	5.91 ± 1.88a	11.31 ± 3.68a	10.4 ± 2.95a	11.41 ± 3.97a			
o-cresol	-	13.9 ± 0	27.05 ± 0	33.35 ± 0	13.15 ± 4	*	****	**
o-cresol	+	2.23 ± 0.93a	8.49 ± 2.5a	7.37 ± 0.6a	5.3 ± 2.41a			
guaiacol	-	2.2 ± 0.93	1.38 ± 0.09	5.57 ± 4.18	3.91 ± 1.58	ns	*	ns
guaiacol	+	6.41 ± 2.64a	12.32 ± 5.21a	12.15 ± 5.02a	10.84 ± 5.28a			
eugenol	-	1.86 ± 0.12	2.01 ± 0.26	2.13 ± 0.23	1.98 ± 0.21	ns	*	ns
eugenol	+	1.68 ± 0.21a	1.63 ± 0.06a	1.68 ± 0.17a	1.56 ± 0.15a			
4-methylguaiacol	-	1.1 ± 0.21	2.1 ± 1.23	2.23 ± 1.26	1.61 ± 0.45	ns	ns	ns
4-methylguaiacol	+	1.76 ± 0.69a	3.43 ± 1.15a	2.54 ± 0.82a	2.54 ± 1.06a			
4-ethylphenol	-	2.01 ± 0.09	2.24 ± 0.45	2 ± 0.13	2.17 ± 0.17	ns	ns	ns
4-ethylphenol	+	0.97 ± 0.27a	2.27 ± 0.59a	1.69 ± 0.32a	2.28 ± 0.72a			
4-ethylguaiacol	-	0.68 ± 0.03	0.87 ± 0.21	0.89 ± 0.23	0.76 ± 0.06	ns	**	ns
4-ethylguaiacol	+							

Notes: [1] Mean VP concentrations (n = 4) are recorded in ng/g ± the standard error of the mean (SEM). [2] The effects of smoke (S), biofilm treatments (T) and S x T interaction were tested with a two-way ANOVA; *, **, *** and **** denote significant differences of $p \leq 0.05$, $p \leq 0.01$, $p \leq 0.001$ and $p \leq 0.0001$, respectively; ns = not significant ($p \geq 0.05$). [3] Different letters (within the rows) indicate the differences in effects of time of biofilm application on the smoked sample only using one-way ANOVA followed by a Tukey's HSD test. Blank cells denote concentrations below the method limit of detection.

Table S7. Vineyard 2: Total (*i.e.* quantitated after acid hydrolysis) VP concentrations immediately after (T_1) smoke-exposure and at harvest (T_2).(1)(2)

VP	smoke	**biofilm application:** days before smoke exposure				S x T	smoke (S)	treatment (T)
		**control**	**1**	**7**	**14**			
**time:** 1 hour post-smoke exposure (T_1)								
syringol	-	8.16 ± 1.82	8.15 ± 0.87	7.01 ± 0.39	6.55 ± 0.84	ns	ns	ns
syringol	+	16.88 ± 7.77a	9.34 ± 0.46a	7.57 ± 1.01a	9.16 ± 1.3a			
phenol	-	27.61 ± 5.75	23.84 ± 2.59	29.62 ± 3.61	22.54 ± 7.82	ns	****	ns
phenol	+	52.88 ± 14.75a	120.72 ± 15.29a	73.85 ± 17.19a	91.11 ± 23.05a			
p/*m*-cresol	-	337.61 ± 76.01	339.31 ± 33.78	426.63 ± 58.74	432.66 ± 211.77	ns	***	ns
p/*m*-cresol	+	90.54 ± 4.65a	97.72 ± 14.87a	68.06 ± 4.41a	98.21 ± 15.95a			
o-cresol	-	3.29 ± 1.65	8.12 ± 3.59	2.35 ± 0.98	6.18 ± 1.52	ns	***	ns
o-cresol	+	13.82 ± 1.26a	44.89 ± 11.72a	32.04 ± 10.22a	58.92 ± 21.86a			
guaiacol	-	3.79 ± 0.32	4.34 ± 0.44	5.15 ± 0.6	3.01 ± 0.62	ns	****	ns
guaiacol	+	17.11 ± 4.41a	72.31 ± 18.81a	41.1 ± 5.79a	59 ± 24.21a			
eugenol	-	3.76 ± 1.23	4.54 ± 0.97	4.31 ± 0.82	3.64 ± 0.4	ns	ns	ns
eugenol	+	3.36 ± 0.81a	4.98 ± 1.21a	3.55 ± 0.57a	4.81 ± 1.6a			
4-methylguaiacol	-		9.79 ± 8.79	4.12 ± 0	8.98 ± 6.07			
4-methylguaiacol	+	7.83 ± 2.4a	26.67 ± 7.92a	9.15 ± 3.2a	27.81 ± 11.47a			
4-ethylphenol	-	15.48 ± 4.92	13.1 ± 1.05	12.1 ± 0.79	12.86 ± 3.26	ns	ns	ns
4-ethylphenol	+	13.59 ± 1.7a	17.51 ± 0.76a	14.59 ± 0.3a	17.95 ± 3.01a			
4-ethylguaiacol	-	2.79 ± 0.32	2.52 ± 0.69	2.38 ± 0.37	1.56 ± 0.24	ns	**	ns
4-ethylguaiacol	+	2.41 ± 0.33a	6.08 ± 1.36a	4.7 ± 0.93a	6.13 ± 2.06a			
**time:** harvest (T_2)								
syringol	-	16.55 ± 2.53	17.58 ± 1.35	15.58 ± 1.15	11.76 ± 0.74	*	ns	ns
syringol	+	13.8 ± 0.66a	12.46 ± 2.78a	14.87 ± 1.13a	16.06 ± 1.09a			
phenol	-	62.2 ± 21.79	42.16 ± 2.53	52.08 ± 3.87	142.96 ± 59.59	ns	*	ns
phenol	+	144.48 ± 53.4a	189.76 ± 85.61a	122.96 ± 77.35a	260.53 ± 213.48a			
p/*m*-cresol	-	19.86 ± 5.38	38.23 ± 22.87	37.14 ± 18.3	37.98 ± 11.94	ns	****	ns
p/*m*-cresol	+	122.9 ± 16.45a	131.28 ± 37.9a	139.96 ± 14.94a	148.18 ± 18.74a			
o-cresol	-	19.11 ± 7.89	15.1 ± 6.99	33.03 ± 24.43	26.14 ± 2.61	ns	ns	ns
o-cresol	+	17.51 ± 5.52a	29.95 ± 9.29a	36.77 ± 14.54a	76.88 ± 20.91a			
guaiacol	-	14.53 ± 4.15	28.25 ± 15.88	29.3 ± 16.31	21.98 ± 6.46	ns	*	ns
guaiacol	+	28.3 ± 8.03a	51.22 ± 17.55a	48.67 ± 17.85a	46.27 ± 14.93a			
eugenol	-	9.7 ± 1.74	10.57 ± 2.33	9.68 ± 0.87	9.95 ± 1.77	ns	****	ns
eugenol	+	4.15 ± 0.48a	4.04 ± 0.73a	3.12 ± 0.83a	3.68 ± 1.04a			
4-methylguaiacol	-	3.34 ± 1.36	2.25 ± 0.36	8.96 ± 6.5	6.2 ± 2.41	ns	**	ns
4-methylguaiacol	+	13.26 ± 1.02a	43.96 ± 21.13a	24.06 ± 12.37a	17.92 ± 4.75a			
4-ethylphenol	-	12.33 ± 1.52	16.16 ± 3.65	15.49 ± 2.47	15.69 ± 1.45	ns	****	ns
4-ethylphenol	+	28.97 ± 6.22a	29.35 ± 6.15a	31.36 ± 5.31a	39.52 ± 3.91a			
4-ethylguaiacol	-	4.63 ± 0.2	5.09 ± 0.69	5.92 ± 0.87	5.77 ± 0.18	ns	ns	ns
4-ethylguaiacol	+	5.2 ± 0.35a	5.06 ± 0.61a	5.16 ± 0.51a	6.12 ± 0.44a			

Notes: (1) Mean VP concentrations (n = 4) are recorded in ng/g ± the standard error of the mean (SEM). (2) The effects of smoke (S), biofilm treatments (T) and S x T interaction were tested with a two-way ANOVA; *, **, *** and **** denote significant differences of $p \leq 0.05$, $p \leq 0.01$, $p \leq 0.001$ and $p \leq 0.0001$, respectively; ns = not significant ($p \geq 0.05$). (3) Different letters (within the rows) indicate the differences in effects of time of biofilm application on the smoked sample only using one-way ANOVA followed by a Tukey's HSD test. Blank cells denote concentrations below the method limit of detection.

Table S8. Vineyard 3: Free VP concentrations immediately after (T_1) smoke-exposure and at harvest (T_2).[(1)(2)]

VP	smoke	**biofilm application:** days before smoke exposure				S x T	smoke (S)	treatment (T)
		**control**	**1**	**7**	**14**			
				**time:** 1 hour post-smoke exposure (T_1)				
syringol	-	0.98 ± 0.02	0.97 ± 0.02	0.96 ± 0	1.02 ± 0.04			
syringol	+	1.01 ± 0.05a	0.99 ± 0.03a	1.01 ± 0.05a	1.06 ± 0.03a	ns	ns	ns
phenol	-	2.76 ± 0.33	2.51 ± 0.27	4.91 ± 1.45	2.81 ± 0.19			
phenol	+	21.36 ± 4.69a	34.49 ± 11.71a	27.88 ± 11.05a	26.96 ± 5.55a	ns	****	ns
p/*m*-cresol	-	0.39 ± 0.13	0.42 ± 0.06	1.13 ± 0.41	0.8 ± 0.32			
p/*m*-cresol	+	11.8 ± 0.95a	24.72 ± 4.51a	18.83 ± 1.56a	21.9 ± 3.17a	ns	***	ns
o-cresol	-	11.05 ± 4.34	7.51 ± 1.11	15.2 ± 3.94	7.05 ± 1.63			
o-cresol	+	17.98 ± 3.31a	22.24 ± 5.2a	20.17 ± 1.12a	22.06 ± 2.12a	ns	***	ns
guaiacol	-	0.66 ± 0.15	0.88 ± 0.09	0.59 ± 0.09	0.56 ± 0.12			
guaiacol	+	19.69 ± 3.75a	39.45 ± 9.56a	29.32 ± 2.37a	34.46 ± 8.37a	ns	****	ns
eugenol	-	1.17 ± 0.02	1.1 ± 0.01	1.25 ± 0.02	1.11 ± 0.01			
eugenol	+	1.11 ± 0.01a	1.57 ± 0.12b	1.47 ± 0.07ab	1.55 ± 0.07b	**	****	*
4-methylguaiacol	-	0.11 ± 0.06	0.2 ± 0.05	0.34 ± 0.04				
4-methylguaiacol	+	8.45 ± 2.06a	16.72 ± 3.94a	12.63 ± 2.37a	15.97 ± 4.32a			
4-ethylphenol	-	1.57 ± 0.15	1.51 ± 0.12	1.39 ± 0.05	1.57 ± 0.12			
4-ethylphenol	+	3.7 ± 0.58a	6.45 ± 0.74a	5.07 ± 0.25a	5.96 ± 0.79a	*	****	*
4-ethylguaiacol	-			0.05 ± 0.04				
4-ethylguaiacol	+	1.14 ± 0.38a	2.87 ± 0.68a	1.77 ± 0.23a	2.46 ± 0.68a			
				**time:** harvest (T_2)				
syringol	-					-	-	-
syringol	+	0.08 ± 0.01a	0.08 ± 0.01a	0.14 ± 0ab	0.15 ± 0.03b			
phenol	-	10.31 ± 0.63	9.63 ± 0.35	9.35 ± 0.3	9.36 ± 0.16	*	****	*
phenol	+	58.02 ± 9.2a	65.82 ± 2.96a	83.35 ± 9.25a	90.13 ± 13.89a			
p/*m*-cresol	-	1.39 ± 0.1	1.32 ± 0.05	1.37 ± 0.06	1.27 ± 0.07	ns	****	ns
p/*m*-cresol	+	14.73 ± 2.54a	17.11 ± 1.53a	17.69 ± 1.05a	20.85 ± 2.54a			
o-cresol	-	10.17 ± 1.35	11.22 ± 0.69	10.43 ± 1.13	9.24 ± 0.59	****	****	****
o-cresol	+	10.38 ± 0.64a	12.02 ± 0.8a	22.73 ± 1.37b	30.76 ± 3.84b			
guaiacol	-	0.68 ± 0.04	0.64 ± 0.07	0.52 ± 0.05	0.53 ± 0.03	ns	****	ns
guaiacol	+	9.47 ± 1.35a	14.47 ± 1.73a	12.29 ± 1.44a	12.89 ± 1.94a			
eugenol	-	0.95 ± 0.23	0.6 ± 0.08	0.34 ± 0.1	0.28 ± 0.05	ns	****	***
eugenol	+	0.61 ± 0.06a	0.53 ± 0.04a	1.18 ± 0.05ab	1.45 ± 0.33b			
4-methylguaiacol	-	0.23 ± 0	0.2 ± 0.01	0.17 ± 0.01	0.19 ± 0.01	ns	****	ns
4-methylguaiacol	+	2.84 ± 0.51a	4.25 ± 0.46a	4.06 ± 0.33a	4.25 ± 0.7a			
4-ethylphenol	-	1.15 ± 0.03	1.08 ± 0.02	1.11 ± 0.05	1.04 ± 0.03	ns	**	ns
4-ethylphenol	+	1.94 ± 0.36a	2.37 ± 0.55a	1.17 ± 0.13a	1.61 ± 0.23a			
4-ethylguaiacol	-	0.05 ± 0.01	0.06 ± 0	0.07 ± 0.02	0.06 ± 0.01	*	****	ns
4-ethylguaiacol	+	0.86 ± 0.1a	0.97 ± 0.08a	0.68 ± 0.03a	0.72 ± 0.08a			

Notes: (1) Mean VP concentrations (n = 4) are recorded in ng/g ± the standard error of the mean (SEM). (2) The effects of smoke (S), biofilm treatments (T) and S x T interaction were tested with a two-way ANOVA; *, **, *** and **** denote significant differences of $p \leq 0.05$, $p \leq 0.01$, $p \leq 0.001$ and $p \leq 0.0001$, respectively; ns = not significant ($p \geq 0.05$). (3) Different letters (within the rows) indicate the differences in effects of time of biofilm application on the smoked sample only using one-way ANOVA followed by a Tukey's HSD test. Blank cells denote concentrations below the method limit of detection.

Table S9. Vineyard 3: Total (*i.e.* quantitated after acid hydrolysis) VP concentrations immediately after (T_1) smoke-exposure and at harvest (T_2).[(1)(2)]

VP	smoke	biofilm application: days before smoke exposure				S x T	smoke (S)	treatment (T)
		control	1	7	14			
				**time:** 1 hour post-smoke exposure (T_1)				
syringol	-	7.42 ± 3.65	6.38 ± 0	13.2 ± 0	3.96 ± 1.38	ns	*	ns
syringol	+	6.45 ± 0a	2.72 ± 1.23a	3.18 ± 0.94a	3.03 ± 0.7a			
phenol	-	44.86 ± 2.9	49.56 ± 2.58	47.28 ± 7.05	34.32 ± 6.29	ns	****	ns
phenol	+	112.55 ± 11.27a	141.86 ± 26.2a	119.98 ± 20.8a	114.55 ± 17.94a			
p/*m*-cresol	-	120.98 ± 18.58	81.13 ± 17.83	111.4 ± 12.54	70.95 ± 29.54	ns	ns	ns
p/*m*-cresol	+	133 ± 27.97a	102.11 ± 10.07a	102.73 ± 6.75a	99.8 ± 6.71a			
o-cresol	-	64.52 ± 9.71	75.24 ± 16.71	55.87 ± 16.7	35.25 ± 10.51	ns	ns	*
o-cresol	+	80.23 ± 11.45a	48.98 ± 16.73a	40.11 ± 6.61a	33.09 ± 4.9a			
guaiacol	-	4.73 ± 0.62	4.97 ± 0.13	3.62 ± 0.26	4.87 ± 0.63	ns	****	ns
guaiacol	+	21.45 ± 2.19a	47.71 ± 14.2a	32.05 ± 1.42a	39.67 ± 8.82a			
eugenol	-		0.47 ± 0.37		0.43 ± 0.1			
eugenol	+	0.39 ± 0.00a		0.39 ± 0.32a	0.43			
4-methylguaiacol	-	283.46 ± 72.35	303.72 ± 86.05	102.81 ± 53.03	244.32 ± 78.03	ns	ns	ns
4-methylguaiacol	+	263.82 ± 35.79a	193.35 ± 105.32a	207.18 ± 65.16a	328.56 ± 58.4a			
4-ethylphenol	-	68.1 ± 11.68	59.7 ± 5.12	50.84 ± 17.77	28.17 ± 11.35	ns	ns	ns
4-ethylphenol	+	81.81 ± 20.35a	56.54 ± 9.88a	59.76 ± 7.42a	54.11 ± 6.9a			
4-ethylguaiacol	-	1.74 ± 0.27	2.29 ± 0.18	1.5 ± 0.24	2.43 ± 0.29	ns	****	*
4-ethylguaiacol	+	2.96 ± 0.4a	6.5 ± 1.54a	4.19 ± 0.29a	5.43 ± 0.8a			
				**time:** harvest (T_2)				
syringol	-	11.86 ± 0.24	12.27 ± 1.59	9.65 ± 1.51	9.48 ± 0.53	ns	***	ns
syringol	+	13.8 ± 0.7a	18.77 ± 2.82a	14.34 ± 1.35a	14.17 ± 0.42a			
phenol	-	35.31 ± 2.16	35.14 ± 2.6	34.83 ± 5.09	38.2 ± 2.02	*	****	*
phenol	+	241.46 ± 21.55a	422.92 ± 67.95a	406.36 ± 25.58a	320.43 ± 34.24a			
p/*m*-cresol	-	7.5 ± 0.5	8.93 ± 0.41	8.54 ± 0.65	8.9 ± 0.43	ns	****	ns
p/*m*-cresol	+	65.72 ± 6.97a	109.08 ± 19.71a	108.1 ± 8.21a	89.95 ± 13.34a			
o-cresol	-	65.46 ± 13.25	72.39 ± 24.11	73.46 ± 8.69	64.36 ± 4	ns	***	*
o-cresol	+	76.48 ± 3.24a	151.43 ± 21.73ab	134.71 ± 15.86ab	90.41 ± 9.42b			
guaiacol	-	5.84 ± 0.34	4.89 ± 0.19	6.7 ± 1.49	5.55 ± 0.18	*	****	*
guaiacol	+	41.42 ± 3.57a	57.45 ± 6.76b	57.9 ± 2.04ab	61.91 ± 3.79ab			
eugenol	-	5.57 ± 1.04	4.45 ± 1.23	2.91 ± 0.31	2.84 ± 0.3	*	***	ns
eugenol	+	6.03 ± 0.13a	4.9 ± 0.52a	6.07 ± 0.38a	6.33 ± 0.35a			
4-methylguaiacol	-	0.56 ± 0.08	0.44 ± 0.09	0.41 ± 0.07	0.38 ± 0.02	**	****	**
4-methylguaiacol	+	15.01 ± 1.82a	21.52 ± 2.77b	22.8 ± 0.63ab	25.36 ± 1.95ab			
4-ethylphenol	-	13.34 ± 2.83	13.43 ± 2.45	13.7 ± 2.93	14.88 ± 1.83	*	**	ns
4-ethylphenol	+	14.88 ± 0.69a	26.19 ± 2.29a	23.66 ± 2.08ab	14.83 ± 2.9b			
4-ethylguaiacol	-	3.01 ± 0.26	2.5 ± 0.23	3.35 ± 0.58	2.7 ± 0.28	ns	****	ns
4-ethylguaiacol	+	4.21 ± 0.22a	4.97 ± 0.28a	5.11 ± 0.17a	4.78 ± 0.53a			

Notes: [(1)] Mean VP concentrations (n = 4) are recorded in ng/g ± the standard error of the mean (SEM). [(2)] The effects of smoke (S), biofilm treatments (T) and S x T interaction were tested with a two-way ANOVA; *, **, *** and **** denote significant differences of $p \leq 0.05$, $p \leq 0.01$, $p \leq 0.001$ and $p \leq 0.0001$, respectively; ns = not significant ($p \geq 0.05$). [(3)] Different letters (within the rows) indicate the differences in effects of time of biofilm application on the smoked sample only using one-way ANOVA followed by a Tukey's HSD test. Blank cells denote concentrations below the method limit of detection.

Table S10. Meteorological data collected for the vineyards used in biofilm field studies.

vineyard	conditions	date day/month/year	temperature[1,2] (°C)	max. temperature	min. temperature	precipitation (mm)	mean temperature[3]	total precipitation[3]
**V1**	biofilm; 14 days	29/08/2019	17.2	31.4	8.5	0		
**V1**	biofilm; seven days	05/09/2019	15.9	27.8	9.6	1.8		
**V1**	biofilm; one day	11/09/2019	13.9	23.2	12.1	0.6		
**V1**	smoke exposure (T_1)	12/09/2019	14.2	23.1	9.9	0.4		
**V1**	harvest (T_2)	30/09/2019	4.5	10.0	1.9	0	15.6	36.4
**V2**	biofilm; 14 days	23/08/2019	13.1	20.0	9	0.7		
**V2**	biofilm; seven days	30/08/2019	19.0	30.1	13.9	0		
**V2**	biofilm; one day	05/09/2019	15.9	27.8	9.6	1.8		
**V2**	smoke exposure (T_1)	06/09/2019	16.8	28.9	9.6	0		
**V2**	harvest (T_2)	06/10/2019	3.7	14.2	-0.4	0	14.7	48.3
**V3**	biofilm; 14 days	03/09/2019	16.1	30.1	8.3	0		
**V3**	biofilm; seven days	10/09/2019	14.4	21.6	11.1	2.9		
**V3**	biofilm; one day	16/09/2019	11.6	19.3	10.1	0.2		
**V3**	smoke exposure (T_1)	17/09/2019	12.8	17.3	8.9	3.6		
**V3**	harvest (T_2)	26/09/2019	11.6	17.3	6.9	1.5	16.8	41.0
**V2[4]**	biofilm; seven days	08/24/2018	14.7	29.6	9.2	0		
**V2[4]**	smoke exposure 1	08/31/2018	13.5	25.5	7.9	0		
**V2[4]**	smoke exposure 1	09/02/2018	10.8	25.8	8.5	5.4		
**V2[4]**	harvest	09/14/2018	6.2	12.7	4.6	0.4	15.7	11.6

Notes: [1]All historical weather data collected by the Government of Canada and accessible online at https://climate.weather.gc.ca/historical_data/search_historic_data_e.html; the data obtained were from the Kelowna, BC meteorological station. [2]Temperature at 0700 local time, when vineyard procedures were performed with the exception of sample collection at harvest. [3]Mean temperature and total precipitation between the first spray application (*i.e.* 14-days post smoke-exposure) and harvest. [4]V2 was the site of our initial evaluation of the bioflm spray also using Pinot Noir grapevines as described by Favell *et al.* (2019); biofim and smoke applications were identical although the specific grapevines used differed.

Table S11. Chemical composition of must and wines.

cold soak

treatment		parameter							
**smoke**	**days post-spray**	**°Brix**	**TA[1] (g/L)**	**pH**	**malic acid (g/L)**	**α-amino acids (mg/L)**	**ammonia (mg/L)**	**YAN[2] (mg/L)**	**potassium (mg/L)**
**-**	**control**	20.7	5.0	3.64	3.26	188	61	239	1223
**-**	**1**	21.8	5.0	3.60	3.23	163	47	201	1210
**-**	**7**	20.2	4.9	3.58	3.04	157	44	194	1105
**-**	**14**	20.3	4.9	3.59	3.06	164	50	205	1100
**+**	**control**	21.5	5.2	3.59	3.24	163	50	204	1148
**+**	**1**	21.6	4.9	3.60	2.94	153	43	188	1154
**+**	**7**	21.0	5.2	3.59	2.97	148	42	182	1123
**+**	**14**	20.8	5.3	3.61	3.26	169	53	212	1176

after alcoholic fermentation

**smoke**	**days post-spray**	**alcohol (% v/v)**	**residual sugar (g/L)**	**TA (g/L)**	**pH**	**volatile acidity (g/L)**	**malic acid (g/L)**	**lactic acid (g/L)**	**FC/A_{280}[3] (mg/L)**
**-**	**control**	12.8	0.4	6.0	3.67	0.22	2.62	0.3	44.6
**-**	**1**	13.5	0.4	6.2	3.67	0.27	2.51	0.3	46.7
**-**	**7**	12.7	0.3	6.2	3.60	0.20	2.49	0.3	44.2
**-**	**14**	13.1	0.5	6.0	3.64	0.20	2.49	0.3	46.8
**+**	**control**	13.4	0.4	6.2	3.65	0.25	2.57	0.3	47.8
**+**	**1**	13.7	0.4	6.2	3.63	0.26	2.24	0.3	51.6
**+**	**7**	12.9	0.4	6.2	3.61	0.29	2.45	0.3	44.5
**+**	**14**	13.0	0.3	6.2	3.65	0.21	2.58	0.3	49.3

Notes:. [(1)]TA = titratable acidity; [(2)]YAN = yeast assimilable nitrogen. (3) FC = Folin-Ciocalteu method for determining total polyphenols; A_{280} = ultraviolet absorbance at 280 nm.

Table S12. Concentration of free VPs in must and wine samples.[(1)(2)]

VP	smoke	biofilm application: days before smoke exposure				S x T	smoke (S)	treatment (T)
		control	1	7	14			
				**must**				
syringol	-	1.18 ± 0			1.27 ± 0			
syringol	+							
phenol	-	1.92 ± 0.46	8.25 ± 0.14	5.96 ± 0.24	8.58 ± 0.49			
phenol	+	22.98 ± 0.97a	26.71 ± 11.16a	20.48 ± 8.66a	35.92 ± 0.36a	ns	****	ns
p/*m*-cresol	-		3.82 ± 1.1	4.34 ± 0.4	5.59 ± 1.67			
p/*m*-cresol	+	5.33 ± 1.8a	9.92 ± 0.81a	6.41 ± 2.28a	11.09 ± 4.78a			
o-cresol	-	3.58 ± 0	10.25 ± 4.22	9.31 ± 1.22	12.3 ± 2.21			
o-cresol	+	6.71 ± 4.61a	5.97 ± 2.22a	6.9 ± 0.76a	7.68 ± 1.15a	ns	ns	ns
guaiacol	-		1.24 ± 0.12	1.15 ± 0				
guaiacol	+	4.09 ± 0.23a	7.12 ± 2.63a	7.83 ± 0.01a	7.52 ± 0.01a			
eugenol	-							
eugenol	+							
4-methylguaiacol	-							
4-methylguaiacol	+		2.32 ± 0.04b	2.19 ± 0.19b	1.52 ± 0.12a			
4-ethylphenol	-							
4-ethylphenol	+			1.36 ± 0				
4-ethylguaiacol	-	1.23 ± 0a	1.82 ± 0.04bc	1.47 ± 0.04ab	1.9 ± 0.12c			
4-ethylguaiacol	+							
				**wine**				
syringol	-	4.2 ± 1.22	4.03 ± 0.6	32.2 ± 28.54	4.51 ± 0.26			
syringol	+	3.46 ± 0.18a	3.69 ± 0.09a	193.29 ± 35.81b	4.29 ± 0.31a	***	**	****
phenol	-	4.89 ± 0.26	7.85 ± 0.65	12.93 ± 0.68	6.07 ± 0.29			
phenol	+	37.18 ± 0.03a	71.39 ± 1.53c	57.44 ± 0.68b	70.45 ± 3.46c	****	****	****
p/*m*-cresol	-		2.53 ± 0.27	3.12 ± 0.17	1.47 ± 0.32			
p/*m*-cresol	+	11.38 ± 0.06a	22.1 ± 0.5a	13.49 ± 5.58a	23.84 ± 0.77a			
o-cresol	-	71.76 ± 8.67	20.45 ± 9.63	3.33 ± 0.14	2.18 ± 0.11			
o-cresol	+	32.26 ± 1.68b	15.57 ± 0.47a	12.57 ± 0.54a	16.85 ± 0.17a	***	ns	****
guaiacol	-		2.2 ± 0.44	1.9 ± 0.27				
guaiacol	+	9.08 ± 0.22a	19.06 ± 0.47c	14.51 ± 0.16b	15.41 ± 0.91b			
eugenol	-	1.38 ± 0.26	1.71 ± 0.01	14.84 ± 13.2	1.5 ± 0.04			
eugenol	+	1.43 ± 0.23a	1.61 ± 0.12a	55.9 ± 23.08a	1.57 ± 0.15a	ns	ns	*
4-methylguaiacol	-			1.74 ± 0				
4-methylguaiacol	+	2.24 ± 0.17a	6.47 ± 0.54b	4.74 ± 0.3b	5.44 ± 0.55b			
4-ethylphenol	-		1.62 ± 0.14	1.18 ± 0				
4-ethylphenol	+	2.59 ± 0.05a	2.77 ± 0.03a	2.6 ± 0.05a	2.82 ± 0.19a			
4-ethylguaiacol	-							
4-ethylguaiacol	+							

Notes: [(1)] Mean VP concentrations (n = 4) are recorded in ng/g ± the standard error of the mean (SEM). (2) The effects of smoke (S), biofilm treatments (T) and S x T interaction were tested with a two-way ANOVA; *, **, *** and **** denote significant differences of $p \le 0.05$, $p \le 0.01$, $p \le 0.001$ and $p \le 0.0001$, respectively; ns = not significant ($P \ge 0.05$). Different letters (within the rows) indicate the differences in effects of time of biofilm application on the smoked sample only using one-way ANOVA followed by a Tukey's HSD test. Blank cells denote concentrations below the method limit of detection

Table S13. Concentration of total (free plus acid-labile) VPs in must and wine samples.[(1)(2)]

VP	smoke	**biofilm application:** days before smoke exposure				S x T	smoke (S)	treatment (T)
		**control**	**1**	**7**	**14**			
				**must**				
syringol	-	113.01 ± 14.24	26.34 ± 10.7	18.94 ± 4.26	8 ± 0.18			
syringol	+	37.74 ± 25.57a	21.55 ± 3.43a	12.49 ± 3.51a	9.58 ± 1a	*	*	****
phenol	-	39.27 ± 2.64	147.15 ± 85.21	55.3 ± 3.4	63.64 ± 4.47			
phenol	+	156.38 ± 1.31a	195.52 ± 66.97a	151.52 ± 73.55a	202.13 ± 10.12a	ns	**	ns
p/*m*-cresol	-	11.87 ± 0.2	37.13 ± 20.44	8.13 ± 5.86	150.15 ± 142.09			
p/*m*-cresol	+	51.74 ± 2.81a	57.27 ± 19.64a	52.09 ± 12.54a	35.11 ± 1.31a	ns	ns	ns
o-cresol	-	189.3 ± 176.99	46.13 ± 22.23	22.76 ± 3.62	9.91 ± 3.51			
o-cresol	+	39.21 ± 16.85a	66.72 ± 21.91a	167.64 ± 108.43a	57.81 ± 3.35a	ns	ns	ns
guaiacol	-	6.74 ± 0.99	5.33 ± 2.43	1.55 ± 0.1	1.65 ± 0.29			
guaiacol	+	5.89 ± 0.78a	6.82 ± 2.68a	6.22 ± 3.32a	10.97 ± 4.49a	ns	ns	ns
eugenol	-	4.58 ± 0.3	4.77 ± 0.2	4.47 ± 0.12	3.46 ± 0.51			
eugenol	+	4.09 ± 0.19a	4.56 ± 0.24a	3.01 ± 0.56a	4.54 ± 0.18a	*	ns	ns
4-methylguaiacol	-		21.9 ± 19.64					
4-methylguaiacol	+	1.46 ± 0.29a	2.63 ± 0.14a	2.34 ± 1.05a	4.78 ± 0.88a	-	-	-
4-ethylphenol	-	16.02 ± 1.55	21.6 ± 4.83	13.56 ± 6.41	16.25 ± 1.29			
4-ethylphenol	+	16.91 ± 7.15a	23.14 ± 3.13a	10.14 ± 7.2a	16.1 ± 1.77a	ns	**	ns
4-ethylguaiacol	-	2.62 ± 0.63	1.38 ± 0	1.35 ± 0	1.19 ± 0.07			
4-ethylguaiacol	+	2.35 ± 0.14a	1.11 ± 0a	1.7 ± 0a	1.71 ± 0.25a	ns	ns	ns
				**wine**				
syringol	-	9.16 ± 0.05	4.92 ± 3.41	9.05 ± 0.09	6.57 ± 2.62			
syringol	+	8.38 ± 3.63a	12.34 ± 0.53a	12.32 ± 0a	10.41 ± 0.82a	ns	ns	ns
phenol	-	56.51 ± 4.7	67.57 ± 30.77	106.85 ± 6.65	65.7 ± 5.49			
phenol	+	153.76 ± 70.65a	150.58 ± 108.28a	154.51 ± 117.07a	226.83 ± 99.53a	ns	ns	ns
p/*m*-cresol	-	6.47 ± 0.98	12.85 ± 0.17	14.97 ± 0.37	6.66 ± 2.73			
p/*m*-cresol	+	36.28 ± 2.56a	61.82 ± 2.58a	37.39 ± 15.46a	54.4 ± 4.9a	ns	****	ns
o-cresol	-	12.52 ± 0.65	17 ± 7.62	24.54 ± 1.3	14.98 ± 0.95			
o-cresol	+	46.49 ± 21.69a	117.69 ± 14.61a	101.21 ± 11.78a	96.38 ± 14.49a	ns	****	*
guaiacol	-	15.14 ± 1.1	18.18 ± 8.15	23.84 ± 2.18	15.72 ± 0.82			
guaiacol	+	39.29 ± 4.46b	64.69 ± 4.98a	53.19 ± 5.45ab	52.13 ± 5.22ab	ns	****	*
eugenol	-	2.37 ± 0.51	1.87 ± 0.17	1.82 ± 0.09	2.29 ± 0.06			
eugenol	+	2.02 ± 0.22a	1.88 ± 0.47a	1.83 ± 0.37a	1.95 ± 0.16a	ns	ns	ns
4-methylguaiacol	-	1.85 ± 0	2.07 ± 0.09	2.78 ± 0	1.18 ± 0.06			
4-methylguaiacol	+	4.28 ± 2.59b	19.62 ± 0.97a	16.06 ± 0.28b	17.82 ± 1.05b	**	****	**
4-ethylphenol	-	15.52 ± 3.2	19.63 ± 7.02	19.82 ± 6.65	13.37 ± 5.17			
4-ethylphenol	+	19.71 ± 2.43a	39.46 ± 3.97a	19.54 ± 12.71a	31.8 ± 5.88a	ns	*	ns
4-ethylguaiacol	-	3.38 ± 0.26	3.28 ± 0.53	5.62 ± 0.39	5.11 ± 0.19			
4-ethylguaiacol	+	5.09 ± 0.63a	6.94 ± 0.5a	6.17 ± 0.4a	8.64 ± 1.63a	ns	***	*

Notes: [(1)] Mean VP concentrations (n = 4) are recorded in ng/g ± the standard error of the mean (SEM). (2) The effects of smoke (S), biofilm treatments (T) and S x T interaction were tested with a two-way ANOVA; *, **, *** and **** denote significant differences of $p \le 0.05$, $p \le 0.01$, $p \le 0.001$ and $p \le 0.0001$, respectively; ns = not significant ($P \ge 0.05$). Different letters (within the rows) indicate the differences in effects of time of biofilm application on the smoked sample only using one-way ANOVA followed by a Tukey's HSD test. Blank cells denote concentrations below the method limit of detection.

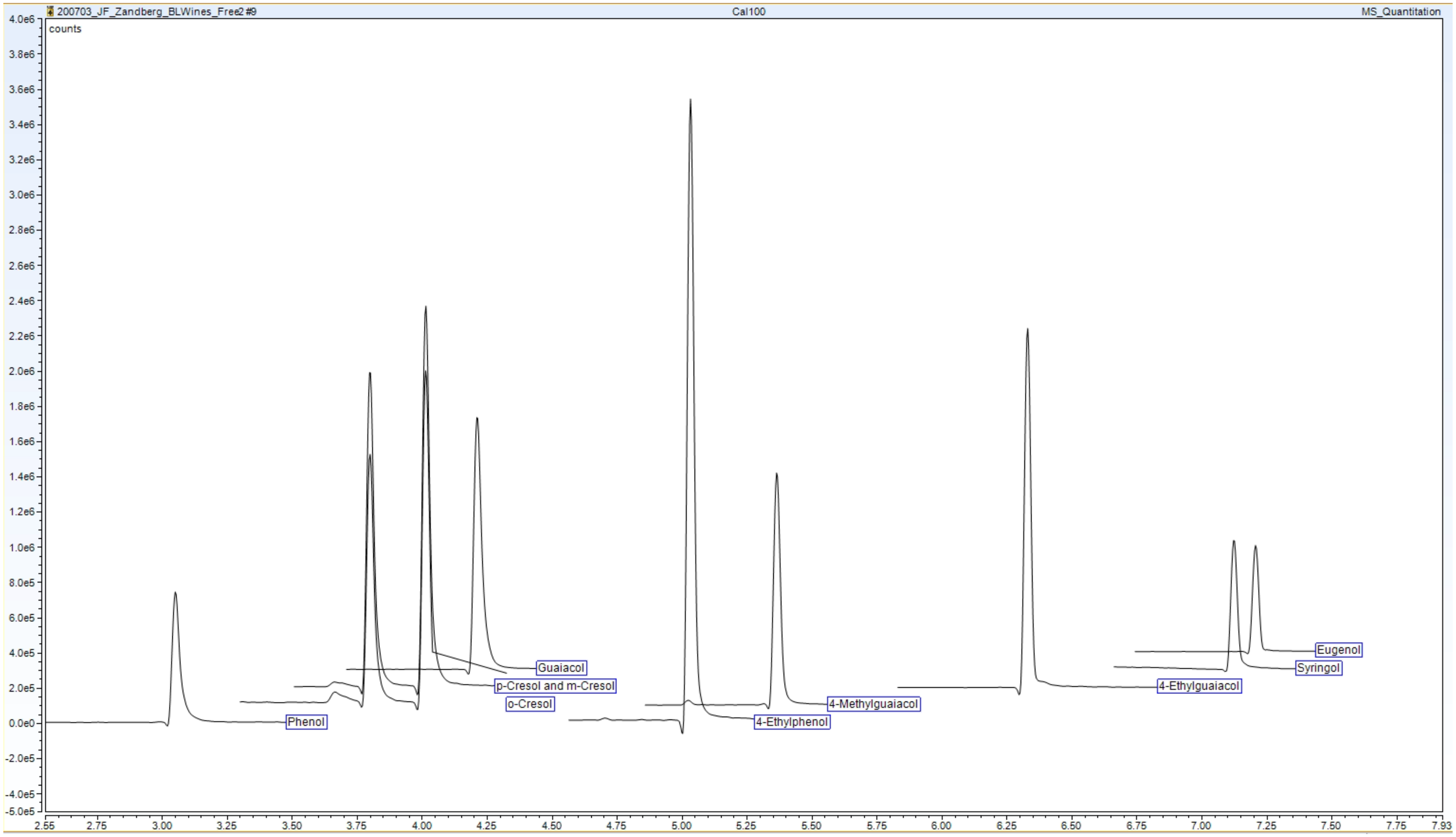

Figure S1. Extracted ion chromatograms for all VPs quantitated.
